# Transcutaneous auricular vagus nerve stimulation: mechanisms, applications, and research progress

**DOI:** 10.3389/fnins.2026.1844063

**Published:** 2026-06-24

**Authors:** Biao-Ping Xu, Xiang-Ming Lin, Ni-Na Lin, Rui Hong, Wen-Ming Lu, Wan-Rong Xu, Ben-Guo Wang

**Affiliations:** 1 Rehabilitation Medicine Department of the Second Affiliated Hospital, School of Medicine, Longgang District People's Hospital of Shenzhen, The Chinese University of Hong Kong, Shenzhen, Guangdong, China; 2 Gannan Medical University, Ganzhou, Jiangxi, China

**Keywords:** autonomic nervous system, clinical application, neuroinflammation, neuromodulation, transcutaneous auricular vagus nerve stimulation, vagus nerve

## Abstract

This review systematically examines the mechanisms of action, optimization of stimulation parameters and targets, and the research progress in the application of taVNS for neurological disorders and systemic conditions. Rather than merely cataloging existing findings, this review critically synthesizes recent advances with a focused emphasis on two core aspects: (1) the mechanistic convergence and divergence among neuroimaging, autonomic, and molecular pathways underlying taVNS effects; and (2) the methodological challenges and biomarker-driven strategies for optimizing stimulation parameters and personalizing treatment. By prioritizing these key directions over exhaustive enumeration of clinical applications, this review aims to provide a conceptually structured framework that distinguishes genuine progress from descriptive accumulation. Moreover, compared with previously published reviews on similar topics, the present work offers a distinctive contribution by integrating multi-modal mechanistic evidence into a testable model of taVNS action and critically evaluating the translational gap between parametric optimization in research settings and standardized clinical implementation. Furthermore, it provides an outlook on future research directions and technological developments, aiming to offer a comprehensive theoretical framework to inform both clinical translation and foundational research in this rapidly evolving field.

## 1 Introduction

Transcutaneous auricular vagus nerve stimulation (taVNS) is a rapidly evolving non-invasive neuromodulation technique that has garnered significant attention over the past two decades (Wang Y. et al., 2021). This method involves the application of electrical current to specific areas of the outer ear, primarily the cymba concha and tragus, which are innervated by the auricular branch of the vagus nerve (ABVN) (Machetanz et al., 2021a; Yamamoto, 2022). By stimulating these cutaneous receptive fields, taVNS aims to indirectly influence central and peripheral nervous system functions, offering a therapeutic alternative to traditional invasive vagus nerve stimulation (VNS) (Wang Y. et al., 2021; Jiakai et al., 2023). Compared to invasive VNS, which requires surgical implantation of a cuff electrode, taVNS offers distinct advantages: non-invasive nature, enhanced safety, lower cost, greater accessibility, and ease of administration. These advantages have substantially broadened its potential application scope (Jiakai et al., 2023; Gerges et al., 2024; Badran, 2022).

The therapeutic potential of taVNS was initially investigated for refractory epilepsy and depression—conditions where invasive VNS has established efficacy. It has since expanded into a diverse array of medical domains (Yamamoto, 2022; Parente et al., 2024; Wang et al., 2022a). A growing body of evidence supports its application in pain management, with studies exploring its efficacy in conditions such as episodic migraine, rheumatoid arthritis, fibromyalgia, irritable bowel syndrome, and chronic low back pain (Chen J. et al., 2025; Zhang J. et al., 2026). For instance, a randomized controlled trial in migraine patients showed that taVNS reduced migraine days and pain intensity, and modulated thalamocortical functional connectivity (Zhang et al., 2021). In the realm of inflammatory and autoimmune diseases, taVNS is thought to engage the cholinergic anti-inflammatory pathway (CAP), offering a neuromodulatory approach to dampen systemic inflammation (Han et al., 2025). Pilot studies have reported benefits in systemic lupus erythematosus (SLE), erosive hand osteoarthritis, and gouty inflammation (Aranow et al., 2021; Courties et al., 2022; Shin et al., 2024). taVNS also shows promise in cardiovascular regulation and metabolic disorders. Research indicates it can increase heart rate variability (HRV) and influence ghrelin, suggesting roles in weight management and glucose tolerance (Machetanz et al., 2021a; Kozorosky et al., 2022; Keute et al., 2021). Its application extends to cognitive enhancement, post-stroke rehabilitation, and other conditions such as tinnitus, disorders of consciousness (DoC), and polycystic ovary syndrome (PCOS) (Chen et al., 2023a; Chen et al., 2023c; Ridgewell et al., 2021; Wu et al., 2024; Briand et al., 2020; Wang et al., 2023; Zhang et al., 2020; Fricke et al., 2025).

Despite this promising expansion, the field faces several core scientific challenges. First, elucidating the precise neurophysiological mechanisms of taVNS. While taVNS activates vagal afferents projecting to the nucleus tractus solitarius (NTS), the subsequent signal propagation is complex and not fully mapped (Owens et al., 2024; Zhang X. et al., 2026). Studies using multimodal neuroimaging (fMRI, EEG) and electrophysiology in both humans and animal models are crucial for delineating these pathways. For example, fMRI studies have shown that taVNS can modulate activity and functional connectivity in networks involving the insula, cingulate cortex, prefrontal regions, and sensorimotor areas, which are implicated in interoception, pain processing, emotion, and cognition (Machetanz et al., 2021a; Poppa et al., 2022; Mao et al., 2022). Second, optimizing stimulation parameters. Factors such as site, laterality, frequency, intensity, pulse width, and duration significantly influence outcomes and may account for interindividual variability (Gerges et al., 2024; Owens et al., 2024; Barbetti et al., 2025; Donovan et al., 2026). Third, establishing reliable biomarkers for taVNS responsiveness. Candidate biomarkers include HRV indices, EEG-derived measures, pupil dilation, and neuroimaging signatures (Keute et al., 2021; Poppa et al., 2022; D'Agostini et al., 2023; Wu et al., 2021). Fourth, advancing high-quality clinical evidence through large-scale, randomized, double-blind, sham-controlled trials with standardized protocols (Zhou et al., 2023; Gerges et al., 2024; Parente et al., 2024). The present review distinguishes itself from previously published works by selectively focusing on the mechanistic convergence across neuroimaging, autonomic, and molecular levels, and by critically addressing the methodological challenges—parameter optimization, biomarker development, and sham control design—that currently limit clinical translation. By prioritizing depth over breadth and mechanism over description, this review aims to provide a conceptually grounded framework to guide future hypothesis-driven research and personalized taVNS protocols.

## 2 Anatomical and physiological basis of taVNS

### 2.1 Distribution and neural pathways of the auricular vagus nerve

**Figure 1:**
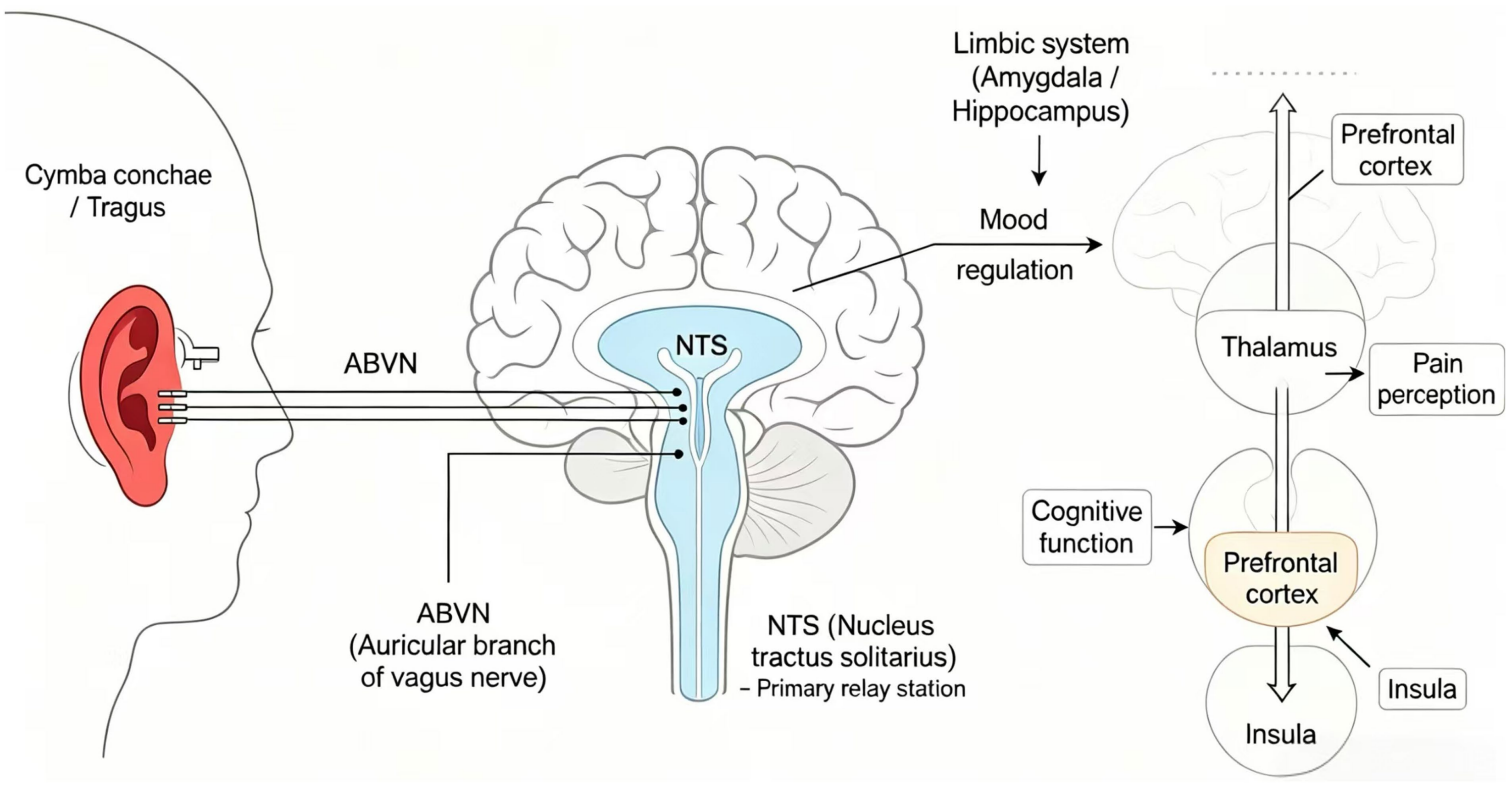
Schematic diagram of the ear-brain pathway in transcutaneous auricular vagus nerve stimulation (taVNS).

The auricular branch of the vagus nerve (ABVN), also known as Arnold’s nerve, represents the only superficial distribution point of the vagus nerve on the body surface, primarily innervating the concha cymba and concha cavum of the external ear, which serves as the anatomical target for taVNS (Wang Y. et al., 2021). This superficial location allows non-invasive access to the vagus nerve, making taVNS a safe and feasible therapeutic option. This non-invasive technique involves applying electrical current to the cutaneous receptive field formed by the ABVN in the outer ear to treat various diseases (Wang Y. et al., 2021). The foundational concept of taVNS is built upon the long-explored connection between the ear and the rest of the body, with its development significantly propelled by theoretical. The stimulation parameters used across taVNS studies show considerable variation. Table 1 summarizes the range of commonly reported parameters in the literature and applied research on ear therapy over the past century (Wang Y. et al., 2021). The stimulation signal from the ABVN is transmitted via afferent fibers to key brainstem nuclei, most notably the nucleus tractus solitarius (NTS) (Owens et al., 2024). The NTS acts as a critical gateway due to its widespread connectivity, relaying and modulating these signals to higher-order brain regions (Owens et al., 2024). From the NTS, the signal ascends through a well-defined neuroanatomical pathway to influence a broad network of brain areas, including the limbic system (such as the amygdala and hippocampus), thalamus, prefrontal cortex, and insula, thereby forming the fundamental “ear-brain” communication pathway (Figure 1) (Briand et al., 2020). This pathway explains how taVNS can influence mood (via the limbic system), pain perception (via the thalamus and insula), and cognitive function (via the prefrontal cortex). This central pathway is central to the proposed therapeutic mechanisms of taVNS, particularly in disorders of consciousness (DOC). Hypothetical mechanism: a hypothesized model, the Vagal Cortical Pathways model, outlines how taVNS may facilitate consciousness recovery through sequential activation of lower and upper brainstem regions, followed by engagement of norepinephrine and serotonin pathways, ultimately impacting cortical and subcortical networks involved in arousal and awareness (Briand et al., 2020). Specific mechanisms may include activation of the ascending reticular activating system, thalamic activation, re-establishment of cortico-striatal-thalamic-cortical loops, and modulation of connectivity between large-scale brain networks like the default mode and salience networks (Briand et al., 2020). Established mechanisms: Functional magnetic resonance imaging (fMRI) studies in healthy humans have demonstrated that taVNS can acutely modulate activity in downstream targets of vagal afferents, including the NTS, substantia nigra, and subthalamic nucleus, with effects that can persist into the post-stimulation period, highlighting its potential to modulate central vagal signaling (Borgmann et al., 2021). Concurrently, taVNS also influences the peripheral nervous system through the efferent fibers of the vagus nerve. This activation impacts peripheral organs such as the spleen, leading to the inhibition of pro-inflammatory cytokine release. This process constitutes the “cholinergic anti-inflammatory pathway,” which is a key mechanism for the anti-inflammatory effects observed with taVNS (Giannino et al., 2024). This pathway underlies the therapeutic potential of taVNS in inflammatory and autoimmune diseases. The anti-inflammatory properties of vagus nerve stimulation, including its non-invasive form, taVNS, are increasingly recognized as crucial for its therapeutic potential in various neurological and psychiatric disorders where neuroinflammation plays a pathogenic role (Zhang X. et al., 2026). Preclinical evidence strongly supports that both invasive cervical VNS (cVNS) and taVNS exert cardioprotective effects in settings like myocardial ischemia/reperfusion injury, mediated through anti-adrenergic, anti-inflammatory, antioxidant, anti-apoptotic, and pro-angiogenic molecular pathways, many of which have been directly confirmed at the cardiac level for taVNS (Giannino et al., 2024). The shared anatomical basis and mechanisms of action between invasive implantable VNS (iVNS) and taVNS underpin the rationale for taVNS as a potential non-invasive alternative, with recent studies revealing similar clinical efficacy for certain indications, thereby potentially expanding the application scope of vagus nerve stimulation (Jiakai et al., 2023). If confirmed, this hypothesis could support taVNS as a treatment for disorders of consciousness. However, direct comparisons at the neuronal level in rats indicate that while taVNS can elicit responses in the NTS comparable to cVNS, it likely does so through distinct neural pathways, as individual neurons show different activation profiles between the two stimulation methods (Owens et al., 2024). Understanding these pathway differences is essential for optimizing taVNS protocols tailored to specific conditions. This underscores the importance of understanding the specific neuronal pathways responsible for taVNS-induced neuromodulation to enable more tailored clinical treatments (Owens et al., 2024).

**Table 1:** Common stimulation parameters reported in taVNS studies.

Parameter	Common variations	Key references
Frequency	1 Hz, 10 Hz, 20 Hz, 25 Hz, 100 Hz, 300 Hz	Owens et al. (2024), Kang et al. (2024), Geng et al. (2022b), Capone et al. (2021), Demircioğlu et al. (2024)
Intensity	0.5–8.1 mA (typically at or above sensory threshold)	Gerges et al. (2024), Owens et al. (2024), Machetanz et al. (2021b), Drost et al. (2025)
Pulse width	100–500 μs (commonly 200–300 μs)	Gerges et al. (2024), Kang et al. (2024), Phillips et al. (2025)
Stimulation site	Cymba conchae, tragus, cavum conchae, earlobe (sham)	Machetanz et al. (2021a), Forte et al. (2022), Matsuoka et al. (2025), Wang R. et al. (2026)
Laterality	Left ear, right ear, bilateral	Barbetti et al. (2025), Zhang et al. (2024a)
Pattern	Continuous, intermittent (burst), phasic	Machetanz et al. (2021a), D'Agostini et al. (2023), Ng et al. (2024)
Duration	Single session (30–60 min), daily (2–4 weeks), long-term (>3 months)	Zhou et al. (2023), Murayama et al. (2025)

### 2.2 taVNS and autonomic nervous system regulation

taVNS exerts a significant modulatory effect on the autonomic nervous system (ANS), primarily characterized by an increase in parasympathetic tone and a concomitant reduction in sympathetic overactivity. This is objectively quantified through physiological metrics such as heart rate variability (HRV) and heart rate (HR). Studies consistently demonstrate that acute or chronic taVNS application leads to an increase in HRV parameters indicative of parasympathetic activity, such as the root mean square of successive differences (RMSSD) and high-frequency (HF) power, while decreasing indices reflecting sympathetic dominance, such as the low-frequency to high-frequency (LF/HF) ratio (Yoshida et al., 2025; Percin et al., 2025; Forte et al., 2022). For instance, during exercise stress tests in healthy volunteers, taVNS significantly reduced HR at peak exercise and during recovery while simultaneously decreasing the LF/HF ratio at rest and during maximum exertion, indicating a shift toward parasympathetic dominance (Yoshida et al., 2025). This parasympathetic enhancement is not merely an acute effect; research in conditions like primary insomnia shows that patients who respond effectively to taVNS exhibit higher HRV parameters (e.g., RMSSD, pNN50, HF) during continuous stimulation compared to non-responders (Wu et al., 2021). Furthermore, the regulation appears to be state-dependent, with evidence suggesting that individuals with baseline low parasympathetic or high sympathetic activity may exhibit a more pronounced response to taVNS, making HRV a potential predictor for treatment responsiveness (Percin et al., 2025; Schiweck et al., 2025). The autonomic effects are also measurable through other means, such as reductions in systolic blood pressure and pulse rate following taVNS sessions (Percin et al., 2025; Perçin et al., 2025). This foundational ability of taVNS to rebalance the ANS away from a sympathetically driven, stress-prone state toward a more parasympathetically mediated, homeostatic condition is a cornerstone of its therapeutic mechanism. However, the evidence is not uniformly positive. A living Bayesian meta-analysis by Wolf et al. (2021) provided strong evidence that acute taVNS does not alter vagally mediated HRV compared to sham in healthy participants, challenging its reliability as a universal acute biomarker. This null finding highlights substantial heterogeneity across studies, which may arise from differences in stimulation parameters (frequency, intensity, site), small sample sizes, baseline autonomic status (individuals with low vagal tone may respond better), and circadian effects (morning > evening). These factors should be systematically addressed in future research to reconcile conflicting results. Recent studies have revealed conflicting HRV findings. Choudhury et al. (2025) reported decreased LF/HF and increased HF, indicating enhanced vagal activity (Choudhury et al., 2025). In contrast, Kaduk et al. (2025) found decreased RMSSD and HF-HRV, suggesting reduced vagal activity (Kaduk et al., 2025). Gianlorenço et al. (2024) showed that age modifies the response, with older adults showing greater HRV increases (Gianlorenço et al., 2024). Atanackov et al. (2025) observed that specific parameters increased SDNN without affecting RMSSD (Atanackov et al., 2025). Critically, Schiweck et al. demonstrated that baseline RMSSD determines response direction: low RMSSD individuals benefit, while high RMSSD individuals show adverse effects (Schiweck et al., 2025). Thus, HRV should be interpreted as a state-dependent or responder-dependent biomarker rather than a universal marker of taVNS efficacy.

The restoration of autonomic balance through taVNS is a core mechanistic pathway underlying its efficacy in treating a wide spectrum of disorders characterized by ANS dysregulation. In psychiatric conditions, the imbalance between sympathetic and parasympathetic systems is closely linked to pathologies like depression and anxiety. taVNS has been shown to mitigate depression induced by insufficient sleep in animal models, an effect accompanied by the restoration of impaired HRV, suggesting its action is mediated through improving vagal function and ANS balance (Ma et al., 2024). For anxiety, particularly in perioperative settings, taVNS is hypothesized to alleviate symptoms by modulating autonomic function and restoring sympathovagal equilibrium (Tang et al., 2025). In cardiovascular contexts, the cardioprotective effects of taVNS are directly linked to parasympathetic activation. In a mouse model of stroke, taVNS attenuated post-stroke cardiac dysfunction, an effect that was abolished by parasympathetic blockade with atropine. This was associated with increased HRV, indicating enhanced parasympathetic and reduced sympathetic dominance (Wang W. et al., 2025). Similarly, in a rat model of Takotsubo syndrome, taVNS alleviated cardiac dysfunction by inhibiting excessive sympathetic activation and systemic inflammation (Jin et al., 2026). For inflammatory and autoimmune diseases, the cholinergic anti-inflammatory pathway, which is vagally mediated, plays a key role. While one review found no direct evidence linking cholinergic anti-inflammatory pathway suppression to postoperative neurocognitive disorders, it acknowledged taVNS as a potential preventive intervention and highlighted the complex role of vagal tone, which may also mediate immunosuppression (Lammers-Lietz et al., 2025). In systemic lupus erythematosus (SLE) models, taVNS retarded disease development and regulated the ANS (Lv et al., 2024). Furthermore, in chronic pain conditions like fibromyalgia, which involves autonomic dysfunction, taVNS effectively reduced pain and improved mood, though specific HRV subparameters (PNS and SNS indices) did not show significant between-group differences in one trial, possibly due to small effect sizes (Akkurt et al., 2025). The broad narrative is that by shifting the ANS toward parasympathetic predominance, taVNS can dampen excessive sympathetic drive, modulate inflammatory reflexes, and promote systemic homeostasis, thereby addressing the pathophysiology of anxiety, insomnia, heart failure, and inflammatory conditions (Croft et al., 2025; Zhang J. et al., 2026).

Research employs a variety of objective biomarkers to quantify the immediate and long-term regulatory effects of taVNS on the ANS, with HRV analysis being the most prominent tool. Time-domain (e.g., SDNN, RMSSD, pNN50), frequency-domain (e.g., HF, LF, LF/HF ratio), and nonlinear HRV parameters provide a noninvasive window into cardiac autonomic control (Soltani et al., 2023; Kang et al., 2024). Studies utilize these metrics to establish “proof of mechanism,” demonstrating that active taVNS, compared to sham stimulation, can induce rapid increases in vagally mediated HRV, particularly when applied to specific auricular sites like the cymba conchae (Katsunuma et al., 2024; Forte et al., 2022). However, the evidence is not entirely uniform, with some meta-analyses of acute taVNS in healthy subjects providing strong evidence for a null effect on vagally mediated HRV compared to sham, highlighting heterogeneity in study designs, stimulation parameters, and participant characteristics (Wolf et al., 2021). This variability underscores the importance of parameter optimization; research indicates that the effects on HRV can depend on the stimulation frequency, pulse width, charge per phase, and anatomical target within the ear (Machetanz et al., 2021b; Atanackov et al., 2025). For example, protocols with 10 Hz/250 μs, 10 Hz/500 μs, and 25 Hz/100 μs were found to increase overall HRV (SDNN) (Atanackov et al., 2025). Beyond HRV, other objective measures include skin conductance (electrodermal activity) as an index of sympathetic arousal (Drost et al., 2025), blood pressure monitoring (Yoshida et al., 2025; Perçin et al., 2025), and analysis of heart rate and baroreflex sensitivity (Soltani et al., 2023). Long-term effects are assessed through repeated measurements over days or weeks of treatment. For instance, in subarachnoid hemorrhage patients, repetitive taVNS increased overall HRV and parasympathetic activity over the treatment course without causing adverse cardiovascular effects. Furthermore, circadian influences on ANS responsiveness have been identified, with morning taVNS sessions producing more significant increases in vagal HRV indices than evening sessions (Geng et al., 2022b). The integration of these objective physiological measures allows for the precise characterization of taVNS-induced autonomic shifts, aids in the identification of optimal stimulation protocols and responsive subgroups, and provides biomarkers for predicting and monitoring therapeutic outcomes across various clinical applications (Wu et al., 2021; Schiweck et al., 2025).

## 3 Stimulation parameters, targets, and device technology of taVNS

This section is divided into three parts: (1) optimization of key stimulation parameters (Section 4.1); (2) precise targeting and verification of stimulation sites (Section 4.2); and (3) device development and technological innovation (Section 4.3).

### 3.1 Research on optimization of key stimulation parameters

Optimizing the stimulation parameters for taVNS is a critical area of research, as these parameters directly influence therapeutic efficacy, safety, and tolerability. The selection of stimulation intensity is foundational, with most protocols employing intensities at or slightly above the sensory threshold to ensure patient comfort while aiming for therapeutic effect (Gerges et al., 2024). However, the relationship between intensity and physiological or clinical outcomes is complex and non-linear. For instance, research indicates that higher intensities, such as 1.0 mA, can elicit greater neuronal responses in brainstem nuclei like the spinal trigeminal nucleus (Sp5) in animal models (Owens et al., 2024). In human studies, specific intensities like 2.0 mA have been shown to induce pupil dilation, a potential biomarker of noradrenergic activation, under scotopic conditions (Capone et al., 2021). Conversely, other findings suggest a non-linear relationship where moderate intensities (e.g., 2–4.8 mA) of high-frequency taVNS can increase pupil size, but this effect may be counteracted at higher intensities (up to 8.1 mA) (Phillips et al., 2025). This underscores the need for personalized dose–response investigations, as the optimal intensity likely varies between individuals and target conditions. The exploration of frequency parameters reveals a similar landscape of variability. While low frequencies (e.g., 20–25 Hz) are commonly used to mimic traditional invasive VNS, evidence suggests that different frequencies may be optimal for different therapeutic goals. In rodent models, both 20 Hz and 100 Hz frequencies were found to generate the highest proportion of neuronal activation in the nucleus of the solitary tract (NTS) and Sp5 (Owens et al., 2024). In clinical contexts, frequency selection appears condition-specific. For example, a frequency of 15 Hz demonstrated superior anti-inflammatory effects compared to 25 Hz in a murine model of endotoxemia (Go et al., 2022). In contrast, for modulating cough sensitivity in a healthy human model, 80 Hz stimulation at the ear canal was more effective than the commonly used 25 Hz at the concha, suggesting parameter adjustments could be crucial for stroke rehabilitation targeting aspiration risk (Ng et al., 2024). The optimization of pulse characteristics, including width and waveform, is another key dimension. Square wave pulses are most frequently employed in taVNS studies (Gerges et al., 2024). The pulse width is often optimized to balance effective nerve fiber recruitment with user comfort, commonly ranging between 200 and 500 microseconds (μs) (Matsuoka et al., 2025). For instance, a home-based trial in Parkinson’s disease utilized specific parameters, including a defined pulse width, which contributed to significant motor and non-motor symptom improvement (Wang R. et al., 2026). The use of biphasic pulses is advocated to minimize net charge accumulation at the electrode-skin interface, thereby reducing the risk of skin irritation and improving long-term tolerability (Gerges et al., 2024). Finally, the stimulation pattern—whether continuous or intermittent—is a subject of active investigation. Continuous stimulation is a standard approach, but intermittent or burst patterns are gaining attention for their potential to better mimic physiological neural firing patterns and enhance neuroplasticity. Studies exploring phasic stimulation (short bursts) have shown it can enhance evoked pupil dilation in a dose-dependent manner, supporting a noradrenergic mechanism (D'Agostini et al., 2023). Furthermore, the specific pattern (interval vs. continuous) has been shown to differentially modulate cognitive event-related potentials like the P300, with continuous stimulation showing larger effects for certain stimuli (Giraudier et al., 2024). The choice of pattern may also influence autonomic outcomes; for example, intermittent bursts of 25 Hz applied at a 1 Hz periodicity have been used to effectively modulate heart rate variability and correlated cortical oscillations (Machetanz et al., 2021a). Overall, the current evidence highlights a significant lack of standardization and comprehensive reporting of taVNS parameters across studies (Gerges et al., 2024). The therapeutic effects of taVNS are demonstrably parameter-dependent, influencing outcomes across neurological, psychiatric, and inflammatory conditions (Matsuoka et al., 2025). Future research must systematically evaluate these key parameters—intensity, frequency, pulse characteristics, and pattern—to establish optimized, condition-specific, and potentially individualized taVNS protocols that maximize efficacy and safety. The stimulation parameters used across taVNS studies show considerable variation. Table 1 summarizes the range of commonly reported parameters in the literature.

### 3.2 Precise targeting and verification of stimulation sites

*After optimizing stimulation parameters, the next critical step is to verify the precise targeting of stimulation sites. Based on anatomy, the cavum conchae and cymba conchae are recognized as the primary target areas. Studies using functional MRI (fMRI) have confirmed that stimulation of this region specifically activates the nucleus tractus solitarius and the limbic system*.

The precise anatomical targeting of taVNS is fundamental to its efficacy and mechanistic validation. The auricular branch of the vagus nerve (ABVN) innervates specific regions of the outer ear, with the cavum conchae and cymba conchae established as the primary target zones due to their dense vagal afferent projections (Wang Y. et al., 2021). Functional magnetic resonance imaging (fMRI) studies provide direct neurophysiological evidence for the specificity of this targeting. Research demonstrates that stimulation applied to the cimba conchae induces significant changes in functional connectivity within key brain networks. For instance, one study found that taVNS alters connectivity in areas involved with emotional processing and regulation, including the limbic regions, insula, frontal lobe, and cerebellum (Monti et al., 2025). This aligns with the proposed pathway where vagal afferents from the ear synapse in the nucleus tractus solitarius (NTS), a major relay station that projects widely to higher brain regions, including the limbic system. Further supporting this, computational modeling of current flow during taVNS confirms that electrode placement over regions like the cymba conchae generates electric fields sufficient for neural activation specifically within that anatomical area, while montages on non-vagal sites like the earlobe restrict significant fields to those control regions (Kreisberg et al., 2021). The engagement of this pathway is not merely anatomical but also has measurable autonomic and clinical correlates. Stimulation of the cimba conchae has been shown to modulate heart rate variability (HRV), a marker of parasympathetic activity, and this effect is correlated with frequency-specific modulation of cortical oscillatory activity, linking peripheral stimulation to central autonomic regulation (Machetanz et al., 2021a). Moreover, targeting the cimba conchae, as opposed to non-vagal sites, has been associated with specific outcomes such as increased pain suppression correlated with parasympathetic activation (Yokota et al., 2024), reduction in inflammatory cytokines in healthy subjects and rheumatoid arthritis patients (Addorisio et al., 2019), and modulation of neural activity in emotion-regulation-related brain regions like the precuneus and temporal gyrus (Zhang et al., 2024b). Therefore, the convergence of anatomical knowledge, neuroimaging evidence, computational modeling, and physiological outcome measures robustly validates the cavum and cymba conchae as the principal target sites for effective and specific vagal efferent engagement via taVNS.

*Non-vagal auricular regions (such as the earlobe) are commonly used as sham stimulation controls to exclude placebo effects and the influence of general somatosensory input*.

The rigorous evaluation of taVNS efficacy necessitates the implementation of controlled study designs that can dissociate specific vagal effects from non-specific consequences of cutaneous electrical stimulation. To this end, stimulation of non-vagal innervated areas of the auricle, most commonly the earlobe, serves as the standard active sham control condition (Wang Y. et al., 2021). This approach is critical for controlling for placebo effects, participant expectations, and the general somatosensory input resulting from the sensation of electrical current on the skin, which can independently influence psychological and physiological measures. The rationale is based on anatomical evidence indicating the earlobe is primarily innervated by the great auricular nerve (cervical plexus) and not the ABVN. Studies consistently employ this methodology to isolate the vagus-specific component of the intervention. For example, research comparing the effects of taVNS at the cymba conchae versus sham stimulation at the earlobe has investigated outcomes across diverse domains, including fear extinction learning (D'Agostini et al., 2025), markers of noradrenergic activity like pupil dilation and salivary alpha-amylase (D'Agostini et al., 2022; D'Agostini et al., 2023), autonomic function (Percin et al., 2024), and cognitive performance during stress (Drost et al., 2025). The use of the earlobe sham allows researchers to match the perceptual experience (e.g., tingling, sensation intensity) between active and control conditions, thereby blinding participants and reducing bias. Computational modeling supports the selectivity of this control, showing that electrode montages on the earlobe generate electric fields concentrated in the earlobe and antitragus, distinct from the vagal targets in the concha (Kreisberg et al., 2021). This distinction is empirically validated by studies showing differential outcomes. For instance, while taVNS at the cymba conchae can modulate brain functional connectivity (Monti et al., 2025) and increase HRV (Forte et al., 2022), earlobe stimulation typically does not produce these specific changes, though it may elicit some autonomic responses under certain protocols, highlighting the importance of the control (Gharabaghi and Keute, 2025). Furthermore, direct comparisons reveal that taVNS at the cymba conchae can elicit more intense sensations than earlobe sham, which must be accounted for in interpretation (D'Agostini et al., 2023). The sham control is also vital in clinical trials, such as those assessing taVNS for postoperative pain, where the sham group receives stimulation at the same ear location (e.g., conchae) but with the device turned off or at a non-effective site to maintain blinding (Huang et al., 2025). Thus, the consistent use of non-vagal auricular sites, particularly the earlobe, as an active control is a methodological cornerstone that strengthens the evidence base for taVNS by ensuring that observed therapeutic effects are attributable to selective vagal pathway activation rather than non-specific confounders.

*Emerging technologies such as individualized auricular 3D modeling combined with electrophysiological navigation aim to achieve more precise and reproducible electrode placement*.

Advancements in technology are paving the way for enhanced precision and personalization in taVNS application, addressing the challenges of inter-individual anatomical variability and inconsistent electrode contact. The development of individualized auricular three-dimensional (3D) modeling represents a significant step forward. This approach can be informed by high-resolution imaging techniques. For instance, high-resolution episcopic imaging (HREM) has been used to create detailed 3D reconstructions of vascular and nerve structures within the human auricular cymba conchae at a micrometer scale, providing a foundational anatomical atlas for optimizing stimulation targeting (Dabiri et al., 2020). Building upon such anatomical data, computational modeling based on individual MRI scans allows for the simulation of electric field distributions during taVNS. One study developed a high-resolution anatomical model of the ear and surrounding tissues from MRI to compare different electrode designs and montages, predicting the sensitivity (required current) and selectivity (spatial distribution) of stimulation for various auricular regions of interest (Kreisberg et al., 2021). This model demonstrates that current flow patterns are highly specific to electrode placement and size, and that relative targeting is robust across individuals. These computational insights directly inform the design of next-generation electrodes. Emerging fabrication methods are producing soft, dry, and compliant electrodes that can conform to the complex curvature of the ear anatomy, improving comfort and ensuring stable electrical performance without the need for conductive gel, which is critical for consistent and long-term application (Shokurov et al., 2026). Furthermore, the concept of closed-loop systems is emerging, where real-time physiological feedback guides stimulation parameters. The integration of ear-EEG, which can measure brain activity from the ear with portable hardware, with taVNS has been proposed as a potential closed-loop system to modulate cognitive states like attention based on neural signatures (Ruhnau and Zaehle, 2021). While still largely investigational, such systems represent the convergence of precise targeting, real-time verification, and adaptive stimulation. Electrophysiological navigation, potentially using such feedback or detailed individual maps, aims to ensure electrode placement directly over zones of optimal nerve density. Although not yet standard in clinical practice, these technological innovations—spanning from detailed anatomical mapping and computational field modeling to advanced biocompatible electrodes and closed-loop paradigms—collectively aim to transform taVNS from a technique with variable application into a precisely targeted, reproducible, and personalized neuromodulation therapy. This progression is essential for maximizing treatment efficacy, understanding dose–response relationships, and ultimately translating taVNS into reliable clinical practice.

### 3.3 Device development and technological innovation

Beyond parameter optimization and site verification, technological advancements in device development are critical for translating taVNS into clinical practice. The landscape of taVNS devices has undergone a significant transformation, evolving from large, stationary laboratory equipment toward portable, wearable, and even smartwatch-integrated systems that support home-based and long-term therapeutic use (Wang et al., 2022a). This trend toward miniaturization and user-friendliness is driven by the need to reduce patient burden, enable repeated daily sessions, and lower the costs associated with clinic-based delivery, thereby enhancing accessibility and treatment adherence (Black et al., 2023). The development of such portable systems is crucial for the practical application of taVNS across various conditions, including chronic pain management, post-stroke rehabilitation, and neuropsychiatric disorders, where consistent, long-term stimulation is often hypothesized to be beneficial (Chen J. et al., 2025; Shi et al., 2023). Concurrently, there is active research into the development of closed-loop feedback taVNS systems. These advanced systems aim to automatically adjust stimulation parameters in real-time based on continuous physiological feedback signals, such as heart rate variability (HRV), electroencephalogram (EEG) patterns, or respiratory cycles, to achieve truly personalized and optimized therapy (de Faria et al., 2023). For instance, integrating ear-EEG sensors with taVNS in a closed-loop setup has been proposed as a potential application to monitor and modulate attention states dynamically (Ruhnau and Zaehle, 2021). The rationale is that such adaptive systems could maximize therapeutic efficacy by responding to the user’s instantaneous physiological state, potentially improving outcomes in cognitive tasks, pain modulation, and autonomic regulation (Ventura-Bort and Weymar, 2024; Perçin et al., 2025). However, the realization of effective closed-loop taVNS faces challenges, including the need for robust, real-time signal processing algorithms and the identification of reliable, stimulation-sensitive biomarkers for feedback control (de Faria et al., 2023; Gianlorenco et al., 2022).

As taVNS devices advance toward commercialization and widespread clinical and home use, paramount importance is placed on establishing rigorous safety standards, ensuring electromagnetic compatibility (EMC), and utilizing biocompatible materials for long-term wear (Wang et al., 2022a). Safety assessments consistently report that taVNS is generally well-tolerated, with adverse events typically being mild and localized to the stimulation site, such as transient discomfort, itching, or headache (Gerges et al., 2024; Deligiannidis et al., 2022). Nevertheless, comprehensive reporting of these events and the specific stimulation parameters used remains inconsistent, highlighting a need for standardized safety protocols (Gerges et al., 2024). A critical technical safety consideration, especially for research involving concurrent neuroimaging, is the risk of radiofrequency-induced heating of stimulation electrodes during magnetic resonance imaging (MRI). Modified stimulator cables with floating ground traps have been developed and tested to mitigate this risk, ensuring safe simultaneous taVNS and functional MRI (fMRI) use while also improving image quality in critical brain regions (Teckentrup et al., 2025). Furthermore, the materials used for electrodes and device housings must exhibit excellent biocompatibility to prevent skin irritation or allergic reactions during prolonged or repeated application, a necessity for daily home-use devices (Song Y. et al., 2025). The electromagnetic compatibility of these electronic devices is another essential factor, ensuring they do not interfere with other medical equipment and function reliably in various environments. Ultimately, the progression from proof-of-concept prototypes to reliable, certified medical devices requires harmonization of technical standards, comprehensive safety testing, and meticulous design focused on user comfort and durability to facilitate the integration of taVNS into mainstream therapeutic practice (Farmer et al., 2020; Wang et al., 2022a). In summary, the wide variation in stimulation parameters across studies underscores the urgent need for standardized, parameter-specific protocols to maximize therapeutic efficacy and ensure reproducibility across different indications and patient populations.

## 4 Application and efficacy of taVNS in neurological diseases

### 4.1 Research quality consideration

As this is a narrative review, we do not perform formal research grading. To help readers understand the current state of clinical research for different taVNS applications, we summarize below the types of research designs available for each indication based on the literature cited in this review. Table 2 lists the highest level of research available for each indication discussed in this review. In addition, a summary of major clinical applications, key mechanisms, and evidence limitations for each disorder is provided in Table 3.

**Table 2:** Summary of research types for taVNS by indication.

Indication	Research evidence available	Key references
Epilepsy	Multiple RCTs + meta-analysis	Teckentrup et al. (2025), Song Y. et al. (2025), Farmer et al. (2020)
Depression	Multiple RCTs + systematic reviews	Tan et al. (2023), Li et al. (2022a), Austelle et al. (2025)
Migraine	RCT with fMRI	Zhang et al. (2021), Fernández-Hernando et al. (2023)
Chronic pain	RCTs	Aoyagi et al. (2025), Molero-Chamizo et al. (2022), Huang et al. (2023)
Post-stroke rehabilitation	RCTs	Moustafa et al. (2025), Sun et al. (2025), Aderinto et al. (2025)
Inflammatory/autoimmune diseases	Animal studies + pilot RCTs	Han et al. (2025), Shin et al. (2024), Geng et al. (2022b), Cirillo et al. (2022)
Cardiovascular/metabolic diseases	Small-sample RCTs + animal studies	He et al. (2022), Zhang et al. (2024a), Jang and Cho (2022)
Other exploratory indications (IBS, PCOS, tinnitus, long COVID, DoC, etc.)	Case reports + pilot studies	Briand et al. (2020), Li et al. (2022a), Pan et al. (2026), Ning et al. (2025)

**Table 3:** Summary of major clinical applications, key mechanisms, and evidence limitations.

Application	Key mechanisms	Current evidence limitations	Key references
Epilepsy	Modulation of large-scale functional brain networks; LC-NE pathway	Mostly short-term outcomes; limited pediatric data	Teckentrup et al. (2025), Song Y. et al. (2025), Farmer et al. (2020), Zhang Q. et al. (2024), Yang et al. (2023), Liao et al. (2025), von Wrede et al. (2021), Hilz (2022), von Wrede et al. (2022)
Depression and anxiety	Limbic-prefrontal circuitry; monoaminergic neurotransmission; BDNF upregulation; anti-inflammatory pathways	Optimal stimulation parameters unclear; high interindividual variability	Sabers et al. (2021), Ma et al. (2025), Pan L. et al. (2024), Tan et al. (2023), Li et al. (2022a), Li et al. (2022b), Liu C. et al. (2024), Koenig and Vöckel (2024), Zhang H. et al. (2024), Ferreira et al. (2024), Austelle et al. (2025)
Chronic pain and migraine	Central descending pain pathways; LC-NE system; thalamocortical modulation	Small sample sizes; heterogeneous pain conditions	Zhang et al. (2021), Aoyagi et al. (2025), Molero-Chamizo et al. (2022), Lommano et al. (2026), Guo et al. (2020), Shao et al. (2023), Huang et al. (2023), Song et al. (2026), Zhu et al. (2024), Straube and Eren (2021), Fernández-Hernando et al. (2023)
Post-stroke rehabilitation	Cortical plasticity; BDNF signaling; motor-sensory integration	Small trials; optimal timing of stimulation unclear	Moustafa et al. (2025), Sun et al. (2025), Aderinto et al. (2025), Zaehle et al. (2021), Zhi et al. (2024), Wang W. et al. (2024), Gong et al. (2025), Choi et al. (2022), Nazari et al. (2025)
Inflammatory/autoimmune diseases	Cholinergic anti-inflammatory pathway (CAP); α7nAChR; cytokine suppression	Mostly preclinical evidence; few large-scale RCTs	Han et al. (2025), Aranow et al. (2021), Courties et al. (2022), Shin et al. (2024), Kozorosky et al. (2022), Go et al. (2022), Tyler (2025), Song X. et al. (2025), Ye et al. (2026), Jensen et al. (2022), Azabou et al. (2021), Yan et al. (2022), Hesampour et al. (2024b), Cirillo et al. (2022), Hesampour et al. (2024a), Wu et al. (2023), Ru et al. (2023), Laurido-Soto et al. (2025b), Shin et al. (2026), Cui et al. (2025)
Cardiovascular/metabolic diseases	Autonomic balance; vagal tone enhancement; anti-inflammatory effects	Small-sample pilot studies; limited long-term data	Carandina et al. (2021), Chen et al. (2020), Couceiro et al. (2023), Liu et al. (2020), de Moraes et al. (2023), Thal et al. (2025), Förster (2024), Souza et al. (2026)

### 4.2 Epilepsy

As an adjunctive therapy for drug-resistant epilepsy, multiple clinical trials have demonstrated that taVNS can reduce seizure frequency and severity. A systematic review and meta-analysis of nine clinical studies involving 788 patients with epilepsy (PWE) found that taVNS had a significantly higher response rate compared to control treatments (Zhang Q. et al., 2024). This aligns with a randomized, double-blind clinical trial of 150 patients, which reported a significantly higher responder rate (seizure frequency reduction >50%) in the active stimulation group compared to the control group after 20 weeks (Yang et al., 2023). Furthermore, a prospective real-world study of 65 patients with drug-resistant epilepsy (DRE) reported an overall efficacy rate of 61.54% for taVNS (Liao et al., 2025). The antiepileptic effects of taVNS are mediated through modulation of large-scale functional brain networks and the LC-NE pathway (see Section 7 for detailed mechanistic discussion) (von Wrede et al., 2021; Hilz, 2022). Supporting this, studies have shown taVNS can induce immediate and enduring reorganization of global network characteristics in patients with epilepsy (von Wrede et al., 2022). The suppression of abnormal synchronous activity is further suggested by research showing taVNS can effectively and rapidly modulate pathological EEG patterns, such as generalized periodic discharges, in patients with possible electrographic status epilepticus (Sarma et al., 2023).

Research focuses on its differential efficacy across various epilepsy syndromes (such as focal and generalized) and its safety profile in pediatric epilepsy. Investigations reveal that the impact of taVNS on functional brain networks differs between epilepsy types. Subjects with generalized epilepsy exhibited distinct baseline network topological properties compared to those with focal epilepsy or controls, and they demonstrated different taVNS-induced immediate and enduring reorganizations of global network characteristics (von Wrede et al., 2022). This suggests taVNS may have syndrome-specific neuromodulatory effects. Regarding safety in children, while specific large-scale pediatric trials for taVNS are limited within the provided references, neurostimulation is recognized as an option for pediatric drug-resistant epilepsy (Ghosh and Nagarajan, 2025). A prospective study investigating taVNS in 11 pediatric patients with drug-resistant epileptic encephalopathy with spike–wave activation in sleep (EE-SWAS) reported it was a safe and effective non-invasive treatment, with 63.63% of patients being responders and no severe adverse events noted (Dai et al., 2025). The safety profile of taVNS is generally favorable. Common adverse events are typically mild and transient, including ear pain, skin irritation, headache, and sleep disturbance (Lampros et al., 2021; Yang et al., 2023; Yamamoto, 2022). A study on depression in PWE noted that adverse events like ear pain or tinnitus often resolved after reducing stimulation intensity or stopping treatment (Xu et al., 2025). Case series in patients with structural focal epilepsy also reported no significant adverse events (Shiraishi et al., 2024). However, feasibility and long-term compliance can be challenging, with practical usability issues and lifestyle fit being significant factors affecting retention rates in some studies (Sabers et al., 2021).

Electroencephalogram (EEG) power spectrum and functional connectivity are being explored as biomarkers to predict treatment response. Studies indicate that taVNS induces measurable changes in EEG parameters. The meta-analysis by Wang et al. showed that the taVNS group exhibited wider EEG changes than the control group (Zhang Q. et al., 2024). Specifically, research has demonstrated that taVNS can reduce the EEG power spectrum. In a randomized controlled trial on patients with comorbid epilepsy and migraine, taVNS reduced the EEG power spectrum across four frequency bands (delta, theta, alpha, beta) at multiple electrode sites compared to the sham group after 24 weeks (Ma et al., 2025). Furthermore, alterations in specific neural oscillations may serve as markers of taVNS effect. A randomized double-blind study in refractory temporal lobe epilepsy found that active taVNS significantly decreased the power spectral density of the theta frequency band in the frontal midline (Fz channel) during working memory tasks, which was correlated with improved cognitive performance (Pan L. et al., 2024). This suggests frontal midline theta oscillations could be a biomarker for taVNS-mediated cognitive regulation. Beyond power spectrum, functional connectivity derived from EEG is a promising biomarker. A study in pediatric EE-SWAS patients used the weighted phase lag index (wPLI) to assess connectivity and found that a 3-month taVNS course significantly decreased coupling strength in specific brain region pairs across theta, alpha, and beta bands, which was associated with clinical improvement (Dai et al., 2025). These findings collectively underscore that EEG-derived metrics, including spectral power and network connectivity measures, are sensitive to taVNS intervention and hold potential for objectively predicting and monitoring therapeutic response. Key message: taVNS is a safe and effective adjunctive therapy for drug-resistant epilepsy, with responder rates comparable to invasive VNS but with a more favorable safety profile.

### 4.3 Depression and anxiety disorders

taVNS exerts its antidepressant and anxiolytic effects through modulation of limbic-prefrontal circuitry, monoaminergic neurotransmission, BDNF upregulation, anti-inflammatory pathways, and the gut-brain axis (102–111) (see Section 7 for detailed mechanistic discussion).

In clinical practice, taVNS is predominantly investigated as an adjunctive therapy, often combined with antidepressant medications, and shows particular promise for improving symptoms in cases of treatment-resistant depression (TRD). A systematic review and meta-analysis of randomized controlled trials (RCTs) concluded that taVNS is an effective and safe method for alleviating depression scores, with a response rate comparable to antidepressants (ATD) when used as monotherapy (Tan et al., 2023). More compellingly, evidence suggests that the combination of taVNS with ATD may offer comparable efficacy to ATD alone but with fewer side effects, highlighting its value as an adjunct (Tan et al., 2023). This is supported by clinical trials demonstrating that taVNS produces symptom improvement similar to first-line antidepressants like citalopram. A 12-week comparative trial found no significant difference in the reduction of Hamilton Depression Rating Scale (HAM-D17) scores between taVNS and citalopram groups, and taVNS even produced a significantly higher remission rate at weeks four and six (Li et al., 2022a). Another study in patients with depression and chronic pain comorbidity found that taVNS combined with electroacupuncture produced positive effects on depressive and pain symptoms similar to citalopram, lasting for at least 8 weeks (Li et al., 2022b). The adjunctive potential of taVNS is further evidenced in specific patient populations. For post-stroke depression, a double-blind RCT showed that taVNS as an add-on to conventional treatment led to significantly greater reductions in depression scale scores and improvements in daily functioning compared to conventional treatment alone (Liu C. et al., 2024). In patients with epilepsy and comorbid depression, a 12-week taVNS treatment significantly improved depressive and anxiety symptoms (Xu et al., 2025). Case reports also support its use in challenging scenarios, such as in an adolescent with treatment-resistant depression, where taVNS over 7.5 months was beneficial (Koenig and Vöckel, 2024), and in postpartum depression, where an open-label trial of a taVNS device showed significant reductions in depression scores, with high response and remission rates (Deligiannidis et al., 2022). Furthermore, taVNS has demonstrated efficacy in alleviating anxiety symptoms across various conditions. A double-blind RCT in Parkinson’s disease patients with anxiety found that a two-week course of 20/4 Hz taVNS significantly reduced anxiety scores compared to sham stimulation, an effect correlated with increased cortical activation in the left inferior frontal gyrus (Zhang H. et al., 2024). In university students with elevated anxiety, active taVNS significantly reduced Beck Anxiety Inventory scores, masseter muscle activity, and altered pain perception compared to sham (Ferreira et al., 2024). A pilot study on inpatients with comorbid depression and anxiety found that both accelerated and spaced taVNS dosing was safe and feasible and significantly reduced scores on depression and anxiety scales (Austelle et al., 2025). These collective findings underscore the role of taVNS as a viable auxiliary treatment strategy, capable of enhancing therapeutic outcomes, potentially reducing medication burden, and addressing symptoms in complex or refractory cases of depression and anxiety.

Current research is increasingly focused on identifying biomarkers and neuroimaging correlates to predict treatment response and to delineate depression subtypes that may be most responsive to taVNS, often employing functional magnetic resonance imaging (fMRI) and serum markers. Neuroimaging studies are pivotal in uncovering the neural signatures of taVNS response. Research indicates that the antidepressant effect of taVNS is associated with the modulation of large-scale brain networks. For example, an 8-week taVNS treatment in MDD patients modulated the brain’s topological architecture, increasing global efficiency and decreasing characteristic path length in functional networks, changes which were correlated with improvements in Hamilton depression scores (Guo et al., 2024). Furthermore, baseline functional connectivity (FC) patterns may predict treatment efficacy. A machine learning study using resting-state fMRI identified 11 FCs associated with the cortico-striatal-pallidum-thalamic loop, hippocampus, and cerebellum that could predict the reduction rate of HAMD-17 scores following taVNS treatment with significant accuracy (Sun J. et al., 2024). Other studies have sought to identify immediate neural changes post-stimulation that might serve as early biomarkers. In patients with TRD, a single 30-min taVNS session immediately reduced regional homogeneity (ReHo) in the medial orbital frontal cortex (mOFC) and altered its connectivity with the left inferior parietal gyrus and superior marginal gyrus (Ma et al., 2022). Similarly, in first-episode, drug-naïve MDD patients, immediate taVNS decreased ReHo in sensorimotor, limbic, and visual-related brain regions (Yi et al., 2022). Beyond neuroimaging, peripheral physiological and molecular markers are being investigated. Heart rate variability (HRV), a measure of autonomic function, has emerged as a potential stratifier. A study found that the effect of taVNS on emotional and inflammatory stress reactivity was not based on diagnostic group (MDD vs. controls) but was significantly influenced by baseline vagal tone (as measured by RMSSD). Participants with low baseline RMSSD showed a restored cardiac stress response under taVNS, while those with high RMSSD showed an adverse pattern, suggesting RMSSD could guide treatment personalization (Schiweck et al., 2025). Serum biomarkers related to inflammation and neuroplasticity are also key targets. Clinical trials have consistently shown that taVNS reduces pro-inflammatory markers like IL-6 and CRP (Parente et al., 2024; Corrêa et al., 2022), while increasing levels associated with resilience, such as BDNF in PSD patients (Liu C. et al., 2024). The exploration of optimal stimulation parameters is another active area of research to define responsive subtypes. Preclinical work suggests that stimulation frequency is critical; in CUMS model rats, 20 Hz taVNS significantly reversed depression-like behaviors and downregulated HPA axis hyperactivity, whereas 5 Hz and 100 Hz did not (Li S. et al., 2020). This indicates that a “depression subtype” characterized by HPA axis dysregulation may respond optimally to specific taVNS parameters. Finally, research is examining taVNS effects on core psychopathological domains like anhedonia and emotional processing. Acute taVNS was found to improve the accuracy of detecting positive facial expressions across participants (including those with MDD) and to reduce self-reported negative emotional states (Zhao et al., 2025). Furthermore, taVNS acutely increased food liking in MDD patients, particularly in those with higher anhedonia scores, and showed a normalization effect on hedonic responses (Koepp et al., 2025). These converging lines of investigation—using fMRI to map network changes, HRV to assess autonomic baseline, and serum assays to track inflammatory and neurotrophic dynamics—are essential steps toward developing a precision medicine framework for taVNS, enabling the identification of patient subgroups most likely to benefit from this neuromodulation therapy. Key message: taVNS is an effective adjunctive treatment for depression and anxiety, with efficacy comparable to first-line antidepressants and fewer side effects.

### 4.4 Chronic pain and migraine

taVNS demonstrates analgesic efficacy across a spectrum of chronic pain conditions, including neuropathic pain, fibromyalgia, and migraine. Clinical studies have shown that taVNS significantly reduces pain intensity and improves functional outcomes in patients with chronic low back pain (Tavares-Figueiredo et al., 2024; Demircioğlu et al., 2024). Neuroimaging evidence indicates that taVNS modulates functional connectivity between brainstem nuclei and cortical/subcortical regions involved in pain processing (Li Y. et al., 2025; Li T. et al., 2025). This modulation of the descending pain modulation system and reward network is believed to underlie its therapeutic effects (128). In knee osteoarthritis, taVNS improves conditioned pain modulation, suggesting enhanced endogenous pain inhibition (Aoyagi et al., 2025). taVNS also alleviates pain in fibromyalgia, potentially by normalizing vagal control over nociceptive and immune-autonomic functions (Molero-Chamizo et al., 2022; Lommano et al., 2026). The analgesic effect is also linked to anti-inflammatory pathways, as taVNS can suppress pro-inflammatory cytokines like TNF-α (Guo et al., 2020). Thus, by engaging a network spanning the brainstem, limbic system, and cortical pain matrix, taVNS offers a multi-target neuromodulatory approach for chronic pain management (Shao et al., 2023; Zhang J. et al., 2026).

In the preventive treatment of migraine, taVNS has shown promise in reducing monthly headache days, with its mechanism intricately linked to the inhibition of the trigeminovascular system and cortical spreading depression (CSD). Clinical trials report that a 4-week course of taVNS significantly decreases the number of migraine days, attack frequency, and pain intensity compared to sham stimulation (Zhang et al., 2021; Huang et al., 2023). The therapeutic action is mediated through the central vagal pathway, activating brainstem nuclei such as the LC and RN (Huang et al., 2023; Song et al., 2026). Animal studies demonstrate that taVNS alleviates migraine-like behaviors by activating the LC-NE system, inhibiting trigeminovascular transmission (Song et al., 2026). By modulating the LC-NE system, taVNS may suppress neurogenic inflammation and vasodilation in migraine (Zhu et al., 2024). Furthermore, taVNS influences thalamocortical circuits, normalizing aberrant sensory processing (Zhang et al., 2021). The inhibition of CSD, the electrophysiological correlate of migraine aura, is another proposed mechanism. While direct human evidence is limited, the modulation of key brainstem and cortical networks by taVNS likely creates a neural environment less conducive to the initiation and propagation of CSD (Hilz, 2022; Straube and Eren, 2021). These actions collectively contribute to taVNS’s efficacy as a non-pharmacological prophylactic option for migraine (Fernández-Hernando et al., 2023).

The immediate analgesic effects of taVNS can be assessed through changes in pain thresholds and fMRI activation. Quantitative sensory testing reveals that a single session of taVNS can increase pressure pain thresholds (Zhang et al., 2024c; Elsehrawy et al., 2025). For instance, in knee osteoarthritis, taVNS improved PPT not only at the affected knee but also at remote sites (e.g., the elbow), suggesting a generalized enhancement of endogenous pain inhibition (Elsehrawy et al., 2025). The conditioned pain modulation paradigm is also augmented following taVNS, corroborating its central mechanism (Zhang et al., 2024c; Aoyagi et al., 2025). fMRI studies demonstrate that acute taVNS can rapidly alter functional connectivity within pain networks, with sustained activation in the NTS and downstream targets (Borgmann et al., 2021). These fMRI biomarkers serve as tools for evaluating taVNS effects and understanding inter-individual variability (Sacca et al., 2023; Feng et al., 2022). Key message: taVNS is a promising non-pharmacological option for migraine prevention and chronic pain management, with effects mediated through central pain modulation pathways (see Section 7 for detailed mechanistic discussion).

### 4.5 Post-stroke rehabilitation and cognitive impairment

*taVNS combined with rehabilitation training (such as exercise therapy) has been proven to enhance motor cortex plasticity and accelerate the recovery of upper limb motor function after stroke*.

taVNS has emerged as a promising non-invasive neuromodulation strategy to enhance motor recovery when paired with rehabilitation training in stroke patients (Shi et al., 2023). The combination of taVNS with conventional rehabilitation or task-oriented training (TOT) has been shown to significantly improve upper limb motor function in both subacute and chronic stroke patients (Wang M. H. et al., 2024; Li J. N. et al., 2022). Clinical trials demonstrate that taVNS paired with rehabilitation leads to greater improvements in Fugl-Meyer Assessment for Upper Extremity (FMA-UE) scores, Action Research Arm Test (ARAT) scores, and activities of daily living compared to sham stimulation plus rehabilitation (Wang M. H. et al., 2024; Wu et al., 2020). Studies using functional near-infrared spectroscopy (fNIRS) and motor-evoked potentials (MEPs) indicate that taVNS paired with training modulates bilateral cortical excitability, increasing activation in the ipsilesional sensorimotor cortex and contralesional prefrontal cortex (Wang M. H. et al., 2024; Li S. Y. et al., 2025). Research exploring closed-loop systems where taVNS bursts are synchronized with electromyography (EMG)-triggered motor attempts aims to optimize this pairing (Xiao et al., 2024). taVNS also upregulates brain-derived neurotrophic factor (BDNF) and activates the BDNF/cAMP/PKA/p-CREB signaling pathway (Li J. et al., 2020; Moustafa et al., 2025). This multi-faceted approach—modulating cortical excitability, activating neurotrophic pathways, and being delivered in synchrony with rehabilitation—positions taVNS as a potent adjunct therapy for accelerating upper limb motor recovery post-stroke (Sun et al., 2025; Aderinto et al., 2025).

*In studies on Alzheimer’s disease and mild cognitive impairment, taVNS may delay cognitive decline by enhancing cholinergic transmission, reducing neuroinflammation, and improving brain network connectivity*.

Research into taVNS suggests its potential to mitigate cognitive decline in conditions like mild cognitive impairment (MCI) and Alzheimer’s disease. By stimulating auricular vagal afferents, taVNS activates the nucleus tractus solitarius (NTS), which projects to the locus coeruleus (LC), thereby increasing norepinephrine release and influencing cortical networks involved in attention and memory (Zaehle et al., 2021; Zhi et al., 2024). taVNS may also enhance cholinergic transmission (Wang W. et al., 2024), reduce neuroinflammation by modulating microglial activity and decreasing pro-inflammatory cytokines (Gong et al., 2025; Sun et al., 2025), facilitate cerebrospinal fluid (CSF) circulation (Choi et al., 2022), and increase BDNF levels (Moustafa et al., 2025; Nazari et al., 2025) (see Section 7 for detailed mechanistic discussion). Clinical trials in MCI patients show that taVNS can improve Montreal Cognitive Assessment (MoCA) scores, enhance performance on auditory verbal learning and shape trails tests, and improve sleep quality (Wang et al., 2022b). By modulating autonomic balance and cortical excitability, taVNS improves functional connectivity within and between brain regions such as the prefrontal cortex and sensorimotor areas, thereby supporting higher cognitive functions (Li S. Y. et al., 2025; Zhang J. et al., 2025).

*Studies use task-state fMRI and neuropsychological scales to evaluate its effects on learning, memory, and executive function*.

The evaluation of taVNS effects on cognitive domains employs a combination of advanced neuroimaging and standardized neuropsychological assessments. Task-based fMRI has been used to map alterations in brain activation patterns, demonstrating improved memory and attention performance alongside increased neural activity (Zhang H. et al., 2022). Functional near-infrared spectroscopy (fNIRS) shows that taVNS paired with training enhances activation in cognition-motor integration areas like the dorsolateral prefrontal cortex (DLPFC) and premotor cortex (Li S. Y. et al., 2025). Neuropsychological scales, including the Montreal Cognitive Assessment (MoCA), the Mini-Mental State Examination (MMSE), and tests for memory, executive function, and processing speed, have shown significant improvements following taVNS compared to sham stimulation (Wang et al., 2022b; Li Z. D. et al., 2022; Liu W. et al., 2024; Chen et al., 2024). Event-related potentials (ERPs), such as the P300 component, are also used as electrophysiological biomarkers (Zhang J. et al., 2025; Gadeyne et al., 2022). Key message: taVNS is a promising adjunct to post-stroke rehabilitation, enhancing motor recovery and potentially slowing cognitive decline through neuroplasticity and anti-inflammatory mechanisms (see Section 7 for detailed mechanistic discussion).

## 5 Exploration of taVNS application in systemic diseases

### 5.1 Inflammation and autoimmune diseases

Preclinical evidence: taVNS exerts its anti-inflammatory effects primarily via the cholinergic anti-inflammatory pathway (CAP), inhibiting pro-inflammatory cytokines such as TNF-α, IL-6, and IL-1β (Tyler, 2025; Song X. et al., 2025; Han et al., 2025). Preclinical evidence robustly supports this mechanism. For instance, in a mouse model of acute gout induced by monosodium urate crystals, taVNS significantly reduced neutrophil infiltration and the expression of inflammatory cytokines and chemokines in the ankle joint tissue, effects that were blocked by α7nAChR antagonism, confirming the pathway’s specificity (Shin et al., 2024). Similarly, in a lipopolysaccharide (LPS)-induced systemic inflammation model in mice, taVNS promoted CAP activity, which significantly attenuated tissue injuries in the lung, spleen, and intestine while decreasing inflammatory cytokine levels and tissue-infiltrated immune cells (Go et al., 2022). The anti-inflammatory efficacy of taVNS appears to be parameter-dependent; one study demonstrated that stimulation at 15 Hz produced a more pronounced reduction in pro-inflammatory cytokines than stimulation at 25 Hz in an LPS-induced endotoxemia model (Go et al., 2022). This foundational mechanism underpins the therapeutic exploration of taVNS across a spectrum of inflammatory and autoimmune conditions, where dysregulated cytokine production is a central pathological feature (Han et al., 2025; Ye et al., 2026).

Clinical evidence: Pilot studies have reported benefits in SLE, erosive hand osteoarthritis, and gouty inflammation, with reductions in pain, fatigue, and inflammatory cytokine levels (13–15). In RA and SLE patients, taVNS increased HRV parameters (Jensen et al., 2022). A randomized cross-over clinical trial protocol has been established to assess taVNS efficacy in axial spondyloarthritis (Azabou et al., 2021). Preclinical work in a rat model of collagen-induced arthritis (CIA) further supports this application, showing that taVNS alleviated cartilage and bone destruction, reduced osteoclast production, and downregulated the expression of matrix metalloproteinases and the RANKL/OPG ratio in synovial tissue (Yan et al., 2022). In inflammatory bowel disease (IBD), encompassing Crohn’s disease and ulcerative colitis, taVNS has demonstrated model-dependent efficacy. In a mouse model of dextran sulfate sodium (DSS)-induced colitis (ulcerative colitis-like), taVNS improved the disease activity index and histological scores, while locally downregulating colonic pro-inflammatory markers (e.g., IL-1β, TNF-α) and upregulating anti-inflammatory cytokines like TGF-β and IL-10 (Hesampour et al., 2024b). It also showed systemic effects, such as decreasing splenic TNF-α and increasing serum TGF-β (Hesampour et al., 2024b). However, these beneficial effects were not observed in a dinitrobenzene sulfonic acid (DNBS)-induced colitis model (Crohn’s disease-like), highlighting the context-specific nature of the intervention (Hesampour et al., 2024b). The rationale for using VNS in IBD is strengthened by evidence of decreased vagal tone and impaired brain-gut axis communication in patients, making neuromodulation a compelling therapeutic strategy (Cirillo et al., 2022; Hesampour et al., 2024a). Beyond these, taVNS has shown anti-inflammatory effects in other conditions, including mitigating gouty inflammation in mice (Shin et al., 2024), reducing pro-inflammatory cytokines in sepsis patients (Wu et al., 2023), and attenuating inflammation in models of postoperative ileus (Ru et al., 2023), acute ischemic stroke (Laurido-Soto et al., 2025b), and otitis media (Shin et al., 2026).

Given its mechanism of action, there is significant interest in utilizing peripheral inflammatory markers as dynamic, objective biomarkers to monitor the anti-inflammatory efficacy of taVNS in real-time. C-reactive protein (CRP), a classic acute-phase protein synthesized by the liver in response to IL-6, serves as a robust and clinically accessible systemic inflammation marker. A randomized clinical trial in hospitalized COVID-19 patients demonstrated that active taVNS led to a significant reduction in CRP levels compared to sham stimulation (Corrêa et al., 2022). This positions CRP as a viable candidate for tracking taVNS-induced modulation of systemic inflammation. Beyond CRP, a panel of cytokines offers a more granular view of the immune response. Studies consistently show that successful taVNS intervention leads to a characteristic shift in the cytokine profile: a decrease in pro-inflammatory mediators (e.g., TNF-α, IL-6, IL-1β) and an increase in anti-inflammatory cytokines (e.g., IL-4, IL-10) (Song X. et al., 2025; Wu et al., 2023). For example, in an LPS-induced depression model in rats, taVNS alleviated depressive-like behaviors and reversed the LPS-induced elevation of serum pro-inflammatory cytokines (IL-1β, TNF-α, MCP-1) while increasing anti-inflammatory IL-4 and IL-10 (Song X. et al., 2025). Similarly, in a pilot study of sepsis patients, taVNS reduced serum TNF-α and IL-1β while increasing IL-4 and IL-10 levels (Wu et al., 2023). The potential of such biomarkers extends beyond mere monitoring; they may also serve as predictive or efficacy-sensitivity indicators. In a clinical trial for treatment-resistant schizophrenia with negative symptoms, the improvement in symptoms after taVNS was significantly correlated with a reduction in TNF-α levels, suggesting TNF-α could be a biomarker of treatment response (Cui et al., 2025). The integration of these peripheral inflammatory markers with central neuromodulation assessments (e.g., functional MRI, EEG) and clinical scores will be crucial for developing personalized taVNS protocols, optimizing stimulation parameters, and objectively evaluating therapeutic outcomes across various inflammatory and autoimmune disorders (Ye et al., 2026; Han et al., 2025).

### 5.2 Cardiovascular diseases and metabolic syndrome

Clinical evidence: taVNS represents a significant non-invasive neuromodulation strategy with promising therapeutic applications in cardiovascular diseases (CVDs) and metabolic syndrome (MetS). The autonomic nervous system (ANS) dysfunction, characterized by sympathetic overactivity and reduced parasympathetic (vagal) tone, is a well-established feature in these conditions (Carandina et al., 2021). taVNS enhances cardiac vagal tone, rebalancing the sympathovagal axis (Chen et al., 2020). This modulation is crucial, as a predominance of sympathetic activity contributes to the onset and worsening of CVDs, including heart failure (HF), hypertension, and arrhythmias (Carandina et al., 2021). In heart failure, autonomic imbalance creates a vicious cycle where excess sympathetic drive and decreased vagal activity worsen cardiac function and prognosis. Clinical studies have demonstrated that taVNS can improve heart rate variability (HRV) in patients with heart failure, indicating improved autonomic balance (Couceiro et al., 2023). This improvement in vagal tone is associated with benefits for cardiac function and reduced risk of arrhythmias (Liu et al., 2020). In patients with MetS, a pilot study found that 8 weeks of taVNS decreased office blood pressure and heart rate, alongside improved sympathovagal balance (de Moraes et al., 2023). The mechanism also involves activation of the cholinergic anti-inflammatory pathway (Han et al., 2025). Chronic low-grade inflammation is a key driver in both MetS and the cardiovascular complications of type 2 diabetes mellitus (T2DM) (Thal et al., 2025). By reducing systemic inflammation, taVNS addresses this pathophysiological component (de Moraes et al., 2023). This anti-inflammatory effect may improve insulin sensitivity and glycemic control in preclinical models of T2DM (Thal et al., 2025). Studies are exploring taVNS for primary prevention of cardiovascular events (Förster, 2024). including combination with aerobic exercise in hypertension (Souza et al., 2026).

### 5.3 Summary of promising indications and evidence gaps

Most promising systemic indications include IBD (ulcerative colitis-like), RA, MetS, and COVID-19-related inflammation, with consistent positive results from preclinical and early clinical studies; current evidence gaps include lack of large-scale RCTs, insufficient long-term safety data, heterogeneous stimulation parameters, and need for validated biomarkers.

## 6 Research progress on the mechanisms of taVNS

### 6.1 A complete regulatory pathway of taVNS

**Figure 2:**
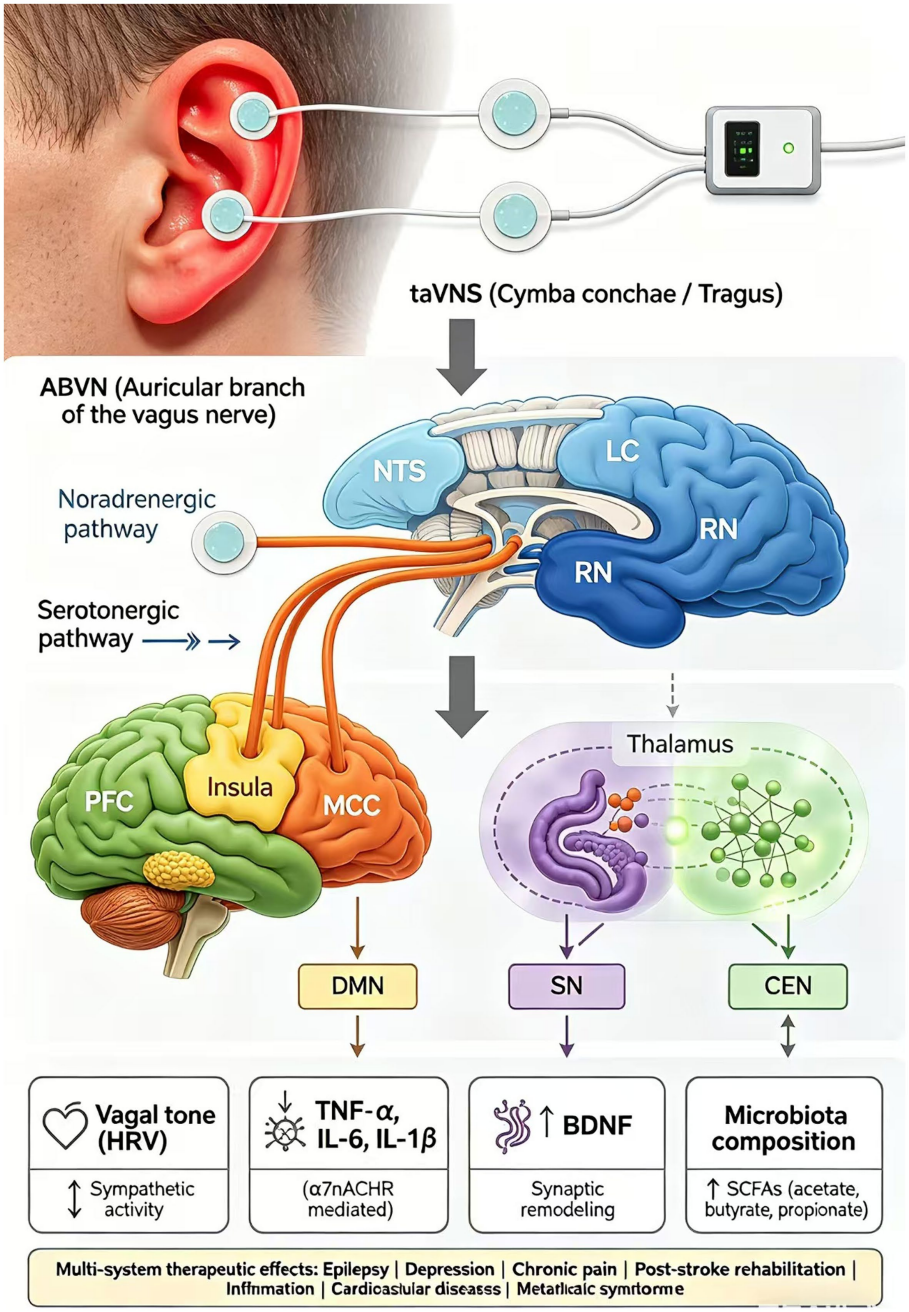
Schematic diagram of the core regulatory mechanisms of taVNS.

Based on the evidence synthesized in this review, taVNS engages a complete regulatory pathway: (1) Peripheral stimulation: electrical current applied to the auricular skin activates the ABVN; (2) Brainstem nuclei: afferent signals are transmitted to the NTS, which projects to the LC, RN, and other brainstem nuclei; (3) Central neural networks: signals ascend to the limbic system (amygdala, hippocampus), thalamus, insula, PFC, and large-scale networks (DMN, SN, CEN); (4) Peripheral target organs: efferent outputs modulate (a) ANS (increased vagal tone, reduced sympathetic activity), (b) CAP (α7nAChR-mediated cytokine suppression), (c) neuroplasticity (BDNF upregulation), and (d) gut-brain axis (microbiota composition, SCFAs). This integrated pathway – auricular skin → ABVN → NTS → central descending pathways / gut-brain axis → peripheral targets – forms the mechanistic foundation of taVNS (Figure 2).

### 6.2 Neuroimaging mechanism studies

Resting-state and task-based functional magnetic resonance imaging (fMRI) studies consistently demonstrate that taVNS modulates functional connectivity within and between major brain networks, including the default mode network (DMN), salience network (SN), and central executive network (CEN). In patients with major depressive disorder (MDD), taVNS treatment has been shown to modulate the brain’s topological architecture, increasing global efficiency and decreasing characteristic path length while reducing connectivity between the DMN, frontoparietal network (FPN), and cingulo-opercular network (CON) (Guo et al., 2024). Predictive models using resting-state fMRI and machine learning have identified specific functional connections associated with the cortico-striatal-pallidum-thalamic loop, hippocampus, and cerebellum that can predict treatment efficacy in MDD (Sun J. et al., 2024). Immediate stimulation in treatment-resistant depression (TRD) modulates regions like the medial orbital frontal cortex (mOFC) and its connectivity with the left inferior parietal gyrus and superior marginal gyrus (Ma et al., 2022). In migraine, taVNS increases connectivity between motor-related thalamic subregions and the anterior cingulate cortex/medial prefrontal cortex while decreasing connectivity between occipital cortex-related thalamic regions and the postcentral gyrus/precuneus, correlating with symptom relief (Zhang et al., 2021). Studies in chronic insomnia show taVNS upregulates activity in the left dorsolateral prefrontal cortex (dlPFC) and decreases its functional connectivity with the bilateral medial prefrontal cortex, with baseline dlPFC activity predicting treatment efficacy (He et al., 2022). Research in healthy subjects confirms taVNS can immediately modulate neural activity, such as reducing regional homogeneity (ReHo) in the precentral and postcentral gyri and altering functional connectivity in different frequency bands (Zhang H. et al., 2026). Furthermore, taVNS modulates functional connectivity between key interoceptive processing regions, notably weakening negative connectivity between the insula and medial prefrontal cortex (MPFC) (Zhang et al., 2024a). In Parkinson’s disease, home-based taVNS improves symptoms and is associated with neuroimaging changes like decreased glutamate in the striatum and thalamus and increased fractional anisotropy in white matter tracts (Wang R. et al., 2026). For disorders of consciousness (DOC), taVNS is associated with positive outcomes and changes in brain activity measured by fMRI and EEG, though research is in early stages (Jang and Cho, 2022). The laterality of stimulation relative to a brain lesion is also critical, as fMRI studies in stroke survivors show ipsilesional taVNS uniquely enhances activation in ipsilesional visuomotor and secondary visual cortex compared to contralesional or bilateral stimulation (Peng et al., 2023). These collective neuroimaging findings across various conditions elucidate that taVNS exerts its therapeutic effects by modulating the functional integration and dynamics of large-scale brain networks involved in executive control, salience detection, self-referential thought, and sensorimotor processing.

Positron emission tomography (PET) studies have revealed taVNS-induced alterations in cerebral neurotransmitter systems, particularly affecting receptor binding rates for gamma-aminobutyric acid (GABA) and serotonin (5-HT), which are central to its neuromodulatory and therapeutic effects. While the provided references primarily utilize fMRI and other modalities, the planned TAVREC trial for patients with prolonged disorders of consciousness (pDoC) explicitly includes positron emission tomography as a secondary outcome measure to investigate taVNS’s neural mechanisms (Zhou et al., 2024). This highlights the recognized potential of PET to elucidate neurochemical changes. The underlying rationale connecting taVNS to neurotransmitter modulation is strongly supported by mechanistic hypotheses and related neuroimaging findings. For instance, the proposed Vagal Cortical Pathways model for DOC suggests taVNS may promote consciousness recovery through serotonergic and noradrenergic pathways originating from the raphe nucleus and locus coeruleus, respectively (Briand et al., 2020). Furthermore, fMRI studies provide indirect evidence of taVNS’s influence on monoaminergic systems. Research in migraine patients demonstrates that repeated taVNS modulates functional connectivity between key brainstem nuclei of the vagal pathway (like the raphe nucleus and locus coeruleus) and cortical/subcortical regions involved in pain and limbic processing (Huang et al., 2023). Changes in connectivity between the raphe nucleus and the putamen were significantly correlated with clinical improvement (Huang et al., 2023). Similarly, in chronic low back pain, taVNS modulates functional and structural connectivity of these same brainstem nuclei (NTS, LC, RN) with widespread cortical regions (Li Y. et al., 2025). The efficacy of taVNS in preventing relapse in remitted MDD is also being investigated with a protocol that includes assessments of serum monoamine neurotransmitters, linking peripheral measures to central effects (Zhang Z. Q. et al., 2022). Although direct PET evidence of receptor binding changes is not detailed in the provided abstracts, these consistent findings across fMRI studies and clinical trial protocols underscore that a primary mechanism of taVNS involves the modulation of central neurotransmitter pathways, including GABAergic, serotonergic, and noradrenergic systems, which are critical for regulating mood, arousal, pain, and consciousness.

Magnetoencephalography (MEG) and electroencephalography (EEG) studies provide direct evidence that taVNS modulates brain oscillatory activity (such as alpha and theta waves) and event-related potentials (ERPs), offering insights into its temporal dynamics and neurophysiological effects. In patients with disorders of consciousness (DOC), studies utilizing EEG have reported positive changes in brain activity following taVNS, contributing to its evaluation as an effective intervention (Jang and Cho, 2022). A specific pilot study in DOC patients used EEG to detect changes in brain connection activity, finding that taVNS differentially regulated delta and beta band energy. In patients with a minimally conscious state (MCS), taVNS induced significant changes in delta and beta band energy across multiple brain regions and altered cross-brain connection activity, suggesting it may improve brain connectivity to aid arousal (Yifei et al., 2022). EEG-derived metrics, including heart-evoked potentials, have also been explored as potential engagement biomarkers for taVNS (Yang et al., 2025). While MEG studies are not explicitly cited in the provided abstracts, the principles of measuring magnetic fields generated by neuronal currents align with the investigation of oscillatory activity. The modulation of brain oscillations is a key proposed mechanism. For example, the efficacy of taVNS in primary insomnia has been linked to its ability to modulate the sensorimotor network, with individual variation in treatment response correlated with autonomic nervous system function as measured by heart rate variability, which is intrinsically linked to central oscillatory rhythms (Wu et al., 2021). Additionally, studies using high-density diffuse optical tomography (HD-DOT), a neuroimaging technology related to functional near-infrared spectroscopy (fNIRS), have demonstrated the feasibility of recording cortical activity during motor tasks with concurrent taVNS, showing reliable task-related hemodynamic responses that can be used to evaluate taVNS effects (Baig et al., 2026). These EEG and related neurophysiological findings collectively indicate that taVNS induces rapid changes in cortical excitability and network synchronization, affecting specific frequency bands and evoked potentials associated with cognitive processing, arousal, and interoceptive awareness, thereby providing a temporal complement to the spatial information obtained from fMRI and PET studies.

### 6.3 Molecular and cellular biological mechanisms

Animal and human studies indicate that taVNS exerts its therapeutic effects through several key molecular and cellular pathways. One primary mechanism involves the upregulation of brain-derived neurotrophic factor (BDNF) expression, which promotes synaptic plasticity and neuronal survival. In animal models, taVNS has been shown to ameliorate depressive- and anxiety-like behaviors, with RNA sequencing analyses revealing that these effects are linked to the regulation of numerous genes in brain regions such as the hippocampus, anterior cingulate cortex, and medial prefrontal cortex (Sun L. et al., 2024). Specifically, in the hippocampus, taVNS appears to enhance glycolysis and suppress inflammatory responses, which are crucial for neuronal health and function (Sun L. et al., 2024). Furthermore, taVNS in anesthetized mice has been demonstrated to induce antidepressant effects by activating dopaminergic neurons in the ventral tegmental area, a process that is dependent on the integrity of this neuronal population and is associated with enhanced neuronal activity (Choi et al., 2024). This activation of specific neural circuits, such as glutamatergic neurons in the anterior cingulate cortex, is also linked to the alleviation of anxiety-like behaviors in models of post-traumatic stress disorder, where taVNS enhances presynaptic excitatory transmission in these activated neurons (Diao et al., 2025). The modulation of gene expression and neuronal activity underscores the role of taVNS in fostering neuroplasticity and resilience.

A second critical mechanism is the attenuation of neuroinflammation through the inhibition of excessive activation of microglia and astrocytes. taVNS has been consistently shown to reduce pro-inflammatory cytokine levels and modulate glial cell states. In a chronic unpredictable mild stress (CUMS) rat model of depression, taVNS reversed depression-like behaviors and hippocampal neuroinflammation, as evidenced by downregulated expression of NF-κB p65 and IL-1β and a shift in microglial morphology from an activated, amoebic-like state to a more quiescent form (Wang J. Y. et al., 2021). This anti-inflammatory effect is mediated, at least in part, by the α7 nicotinic acetylcholine receptor (α7nAChR). Studies using α7nAChR gene knockout rats or antagonists like methyllycaconitine (MLA) have demonstrated that the antidepressant and anti-neuroinflammatory effects of taVNS are significantly diminished when this receptor is blocked, confirming the pathway’s centrality (Wang J. Y. et al., 2021; Chen et al., 2023d). The anti-inflammatory action extends beyond the brain. In a lipopolysaccharide (LPS)-induced depressive-like behavior model, taVNS exerted anti-inflammatory effects by modulating the splenic α7nAChR/JAK2/STAT3 signaling pathway, reducing serum levels of inflammatory chemokines like CXCL1 and increasing anti-inflammatory IL-10 (Chen et al., 2023b). Similarly, in a mouse model of autism spectrum disorder induced by maternal immune activation, taVNS improved social deficits by reducing microglial number, inhibiting M1 polarization, and attenuating the expression of the pro-inflammatory cytokine IL-17a in the prefrontal cortex and blood (Zhang W. et al., 2024). In models of physical injury, such as traumatic brain injury (TBI) and otitis media, taVNS reduced the expression of pro-inflammatory microglial markers (e.g., Tnf, Lcn2) and increased anti-inflammatory markers (e.g., Arg1), while also suppressing NF-κB activation in the middle ear, effects that were abolished by α7nAChR antagonism (El Baassiri et al., 2025; Shin et al., 2026). These findings collectively highlight taVNS’s potent role in modulating central and peripheral immune responses.

A third fundamental mechanism involves the regulation of the hypothalamic–pituitary–adrenal (HPA) axis function and the reduction of cortisol levels, which is particularly relevant for treating stress-related disorders. taVNS influences autonomic balance, promoting parasympathetic predominance, which counteracts the sympathetic hyperactivity and HPA axis dysregulation characteristic of chronic stress. Research shows that taVNS modulates brain-heart interactions, with stimulation-induced increases in heart rate variability (HRV)—a marker of parasympathetic activity—corresponding to frequency-specific oscillatory modulation in cortical areas like the frontal theta-band (Machetanz et al., 2021a). This parasympathetic activation is short-lived but consistent, indicating a direct effect on autonomic outflow (Keute et al., 2021). By enhancing parasympathetic tone, taVNS can mitigate the stress response. In the context of depression, often comorbid with HPA axis hyperactivity, taVNS has been shown to exert antidepressant effects through pathways that involve the regulation of inflammatory and neuroendocrine signals. For instance, in CUMS-exposed rats, the antidepressant-like behavior induced by taVNS was associated with the hypothalamic α7nAChR/JAK2/STAT3/NF-κB signaling pathway, which interfaces with stress and inflammatory responses (Chen et al., 2023d). Furthermore, in a model of depression-chronic pain comorbidity, both taVNS and electroacupuncture reduced pain and depressive behaviors while suppressing elevated levels of the pro-inflammatory cytokine TNF-α in the plasma and brain regions like the prefrontal cortex, hippocampus, amygdala, and hypothalamus (Guo et al., 2020). The hypothalamus, a key regulator of the HPA axis, is a direct target of this modulation. In LPS-induced depressive rats, the antidepressant effect of taVNS was linked to the regulation of the α7nAChR/JAK2 signaling pathway specifically in the hypothalamus and amygdala (Wang J. et al., 2025). By dampening neuroinflammation in these limbic and hypothalamic regions and promoting cholinergic anti-inflammatory pathways, taVNS helps restore HPA axis homeostasis, thereby reducing the physiological impact of stress and forming a core mechanism for its efficacy in stress-related conditions such as depression, anxiety, and PTSD.

### 6.4 Impact on the gut microbiota-brain axis

Emerging evidence suggests that taVNS can influence gastrointestinal motility, intestinal permeability, and the composition of the gut microbiota via vagal pathways, thereby modulating the gut-brain axis. In a mouse model of constipation-predominant irritable bowel syndrome (IBS-C), taVNS treatment was found to increase fecal pellet number, fecal water content, and gastrointestinal transit, indicating improved gut motility (Liu et al., 2024a). Furthermore, a clinical trial in patients with IBS-C demonstrated that taVNS significantly improved constipation symptoms, increased the weekly frequency of spontaneous bowel movements, and enhanced rectal sensory thresholds, effects that were associated with elevated serum acetylcholine levels and enhanced vagal activity as measured by heart rate variability (Liu et al., 2024b). These findings directly link taVNS to vagally-mediated improvements in gastrointestinal function. Beyond motility, taVNS appears to modulate the gut microbial ecosystem. In the IBS-C mouse model, taVNS restored the abundance of beneficial genera such as *Lactobacillus* and increased *Bifidobacterium* (Liu et al., 2024a). Similarly, in patients with IBS-C, taVNS led to a significant rise in the genus *Bifidobacterium* (Liu et al., 2024b). In rodent models of depression induced by chronic unpredictable mild stress (CUMS), taVNS intervention reversed gut microbiota dysbiosis, improving the community structure and modulating specific bacterial groups like *Lachnospiraceae*, *Lactobacillus*, *Tyzzerella*, and *Bacteroides* (Zhang X. et al., 2025). Another study in CUMS rats found that taVNS reshaped the microbiota, notably increasing the abundance of *Akkermansia muciniphila* and *Ligilactobacillus murinus* while reducing *Limosilactobacillus reuteri* and *Lactobacillus johnsonii* (Pan et al., 2026). These alterations in microbial composition are significant as they are posited to influence central nervous system function and inflammatory status, forming a critical part of the bidirectional communication loop.

Changes in gut microbial metabolites, such as short-chain fatty acids (SCFAs), may in turn affect central neural function and inflammatory states, constituting a bidirectional regulatory circuit. SCFAs, produced by microbial fermentation of dietary fiber, are key signaling molecules in the gut-brain axis. The clinical trial in IBS-C patients reported that taVNS significantly increased fecal levels of acetic, butyric, and propionic acids (Liu et al., 2024b). Butyrate, in particular, is known for its anti-inflammatory and neuroprotective properties. This modulation of SCFAs likely represents a mechanism by which taVNS-induced microbial changes exert systemic effects. Furthermore, taVNS in CUMS rats was associated with shifts in plasma metabolomic profiles, with differential metabolites enriched in pathways related to bile acid metabolism, arachidonic acid metabolism, and vitamin digestion and absorption (Zhang X. et al., 2025). Correlation analyses in that study suggested that pathogenic microbial genera were positively correlated with plasma metabolites involved in inflammation and metabolic dysregulation, while beneficial microbiota showed opposite trends, indicating a microbial-metabolic link to peripheral inflammation (Zhang X. et al., 2025). This peripheral metabolic and inflammatory modulation can feedback to the brain. For instance, gut dysbiosis and associated inflammation are recognized contributors to neuroinflammation and demyelination in various psychiatric and neurological disorders, with the vagus nerve being a key mediator (Murayama et al., 2025). The bidirectional nature of this axis is highlighted by evidence that gut-derived metabolites and immune signals can activate the vagus nerve, which then relays information to the brainstem (Liang et al., 2026). Conversely, central states can alter gut function and microbiota, as seen in conditions like stroke, which triggers intestinal dyshomeostasis via the brain-gut axis (Bai et al., 2025). taVNS, by stimulating vagal afferents, may interrupt detrimental feedback loops and promote a shift toward a healthier microbial and metabolic profile, thereby reducing systemic and potentially neuro-inflammation.

This mechanism provides a novel explanation for the therapeutic application of taVNS in treating irritable bowel syndrome, obesity, and neuropsychiatric disorders associated with dysbiosis. For IBS-C, the efficacy of taVNS is linked to its integrative effects on rectal function, vagal activity, cholinergic signaling, and the restoration of a healthier gut microbiome and SCFA production (Liu et al., 2024a,b). In the context of obesity, a condition strongly associated with alterations in the gut-brain axis (Asadi et al., 2022), a proposed randomized controlled trial aims to investigate taVNS’s weight-loss effects by specifically assessing its modulation of the microbiota-gut-brain axis through measures of gut peptides, microbiota composition, and functional brain imaging (Chen X. et al., 2025). This underscores the hypothesis that taVNS’s metabolic benefits may be mediated through this pathway. Most compellingly, the antidepressant effects of taVNS appear to be closely tied to the microbiota-gut-brain axis. In CUMS rats, taVNS alleviated depressive-like behaviors concurrently with regulating gut microbiota and mitigating peripheral inflammation and metabolic disorders (Zhang X. et al., 2025). Multi-omics research revealed that the antidepressant properties of taVNS were associated with the upregulation of hippocampal GluN1/MAPK/BDNF signaling, a pathway crucial for synaptic plasticity, and these neural changes were correlated with specific taVNS-induced shifts in gut microbiota composition (Pan et al., 2026). This suggests that the vagus nerve serves as a conduit for microbial signals to influence brain function. The role of the vagus nerve in this axis is further emphasized in other conditions; for example, dysregulation of the gut-brain axis is implicated in the pathophysiology of Parkinson’s disease, where vagal transmission is thought to facilitate the spread of pathological alpha-synuclein from the gut to the brain (Pan I. et al., 2024; Lei et al., 2021). Similarly, in post-COVID conditions (“long COVID”), dysbiosis and gut-brain axis disruption are suspected contributors to neurological symptoms, with vagus nerve stimulation discussed as a potential therapeutic avenue (Hashimoto, 2023; Iqbal et al., 2025). Therefore, by modulating the gut microbiota-brain axis, taVNS presents a promising non-invasive neuromodulation strategy for a spectrum of disorders rooted in dysfunctional gut-brain communication.

### 6.5 Interactions among autonomic, anti-inflammatory, neuroplastic, and gut-brain mechanisms

The four mechanistic domains interact synergistically. First, increased vagal tone (ANS regulation) directly activates the CAP via efferent vagal fibers, suppressing pro-inflammatory cytokines. Second, reduced neuroinflammation facilitates neuroplasticity (BDNF expression, synaptic remodeling). Third, gut microbiota modulation influences systemic inflammation via microbial metabolites (e.g., SCFAs), which in turn affect CNS function via the vagus nerve (bidirectional gut-brain axis). Fourth, central network modulation (e.g., LC-NE system activation) simultaneously enhances cognition, reduces pain, and regulates autonomic outflow. These interconnected mechanisms explain taVNS’s multi-system therapeutic effects.

## 7 Clinical research design and methodological challenges

### 7.1 Sham control and blinding methods

The design of effective placebo controls and robust blinding methods constitutes a primary challenge in taVNS clinical trials, essential for isolating its specific neurophysiological effects from non-specific placebo and attentional confounds. A fundamental strategy involves the use of anatomically distinct sham stimulation sites, most commonly the earlobe, which is not innervated by the vagus nerve’s auricular branch. For instance, studies on memory, stress, and Parkinson’s disease (PD) have employed sham taVNS at the earlobe, contrasting it with active stimulation at the cymba conchae or tragus (Cibulcova et al., 2024; Sanchez-Perez et al., 2023; Wang R. et al., 2026). This approach aims to provide a credible sensory experience—such as tingling or mild discomfort—without activating the targeted vagal pathway. However, ensuring successful blinding is complex, as differences in perceived sensation between the active vagal site and the sham site can potentially unmask the allocation. Some protocols, like those used in studies on cortical excitability or autonomic function, attempt to mitigate this by initially ramping up the sham stimulation intensity to a perceptible level before reducing it to zero or a sub-sensory level, though participant guesses about group allocation can still be imbalanced (Gerges et al., 2025; Liebano et al., 2024). Beyond site location, the use of “active placebo” or sub-threshold stimulation parameters is another critical design element. Research has utilized high-frequency (e.g., 20 kHz) sub-threshold taVNS, which is perceived but theorized to be below the threshold for consistent neural activation, to study effects on cerebral blood flow and functional connectivity while controlling for the somatosensory experience of stimulation (Mao et al., 2022; Chen et al., 2021). The integrity of blinding is frequently assessed via post-trial questionnaires asking participants to guess their group assignment. While many studies report successful blinding, others note challenges; for example, one trial found 96% of participants could detect active taVNS compared to 22% detecting sham, highlighting the difficulty in creating a perfectly indistinguishable control (Gerges et al., 2025). Furthermore, crossover designs are employed to control for inter-individual variability and separate time effects from treatment effects, as seen in studies on pain thresholds and stress responses (Liebano et al., 2024; Austelle et al., 2024). The development and validation of standardized, objective questionnaires to assess blinding success remain an important unmet need, as current methods rely on subjective participant report which may be influenced by expectation and therapeutic outcome (Gerges et al., 2024). Ultimately, the choice of sham protocol—whether earlobe stimulation, sub-threshold parameters, or a different modality like transcutaneous forearm stimulation as an active control in olfactory studies—must be carefully tailored to the research question, ensuring it adequately controls for placebo effects, patient expectations, and the non-specific attentional components inherent to any therapeutic intervention, thereby allowing for a more accurate evaluation of taVNS’s specific mechanistic and clinical efficacy (Gerges et al., 2024; Weise et al., 2025).

### 7.2 Efficacy evaluation, biomarker exploration, and impact of study design

Beyond relying solely on clinical symptom scales, there is a concerted effort to identify objective and quantifiable biomarkers for evaluating the efficacy of taVNS. Pupillometry has emerged as a promising candidate, with studies demonstrating that phasic taVNS can systematically modulate pupil dilation, a marker linked to locus coeruleus-noradrenergic (LC-NA) system activity (Wienke et al., 2023). Furthermore, the pupillary light reflex has been proposed as a simple and effective proxy for taVNS efficacy (Wienke et al., 2023). Research indicates a non-linear relationship between taVNS intensity and pupil diameter, with specific parameters (e.g., 300 Hz stimulation at the external acoustic meatus) increasing pupil size within a certain intensity range (2–4.8 mA), an effect that may be counteracted at higher intensities (Phillips et al., 2025). Short bursts of taVNS have also been shown to enhance evoked pupil dilation in a dose-dependent manner, supporting a noradrenergic mechanism (D'Agostini et al., 2023). However, the effects on pupil size are parameter-dependent, with significant dilation observed under specific illuminance conditions and at particular stimulation intensities (Capone et al., 2021). While some studies report taVNS-induced increases in tonic pupil size (Capone et al., 2021), others have found no such effect (D'Agostini et al., 2021), highlighting the influence of methodological variations. HRV as a candidate biomarker is discussed in detail in Section 2.2, including the conflicting evidence reported by Wolf et al. (2021), Choudhury et al. (2025), Kaduk et al. (2025), Gianlorenço et al. (2024), Atanackov et al. (2025), and Schiweck et al. (2025), as well as factors contributing to heterogeneity across studies. Neuroimaging biomarkers are also under exploration. Functional MRI studies in primary insomnia disorder (PID) patients suggest that taVNS efficacy is correlated with baseline activity in networks such as the default mode network (DMN) and visual network (VN), and with high-frequency HRV parameters during stimulation (Wu et al., 2025). Furthermore, baseline fractional amplitude of low-frequency fluctuation (fALFF) features in thalamocortical circuits and the DMN can predict treatment response in migraine without aura patients (Feng et al., 2022). In treatment-resistant schizophrenia, changes in negative symptoms following taVNS were correlated with alterations in serum tumor necrosis factor-alpha (TNF-α) levels and beta-band electroencephalographic coherence between the left frontal and parietal regions, identifying them as potential sensitivity biomarkers (Baig et al., 2026). Electroencephalographic markers extend beyond spectral power. taVNS has been shown to modulate the heart-evoked potential (HEP), with effects localized to the insula and connected regions, suggesting it could serve as an objective outcome parameter for cortical effects (Poppa et al., 2022). Additionally, taVNS can enhance short-latency afferent inhibition, an indirect biomarker of central cholinergic system activation (Horinouchi et al., 2024).

The design of a clinical trial profoundly influences the interpretation of its biomarker and efficacy results. For example, small sample sizes increase the risk of false positives and inflated effect sizes; lack of adequate sham control may attribute placebo responses to true biological effects; the timing of biomarker assessment (acute vs. chronic) captures different mechanistic phases; and the choice of statistical model (e.g., adjustment for baseline values) affects outcome significance. Furthermore, studies using different stimulation parameters (frequency, intensity, site) may produce contradictory biomarker patterns, making cross-study comparison difficult without standardized reporting. Therefore, interpretation of taVNS biomarker findings must account for these design elements.

The establishment of predictive biomarker models is crucial for identifying patient populations most likely to benefit from taVNS, thereby advancing precision medicine. Research indicates that individual physiological characteristics at baseline can predict treatment response. For instance, baseline neuro-cardiac coupling scores are significant predictors for the individual effect of taVNS on HRV (Keute et al., 2021). In primary insomnia, the baseline mean amplitude of low-frequency fluctuations (mALFF) in a combination of differentially active brain regions (especially DMN + VN) is correlated with taVNS efficacy (Wu et al., 2025). A support vector regression model has demonstrated that baseline functional connectivity of the basal forebrain with visual cortical regions can predict taVNS treatment response in primary insomnia patients (Qi et al., 2025). Similarly, baseline fALFF features in specific brain networks can predict the clinical outcome of taVNS in migraine patients (Feng et al., 2022). Biomarkers measured during or immediately after stimulation may also have predictive value. In subarachnoid hemorrhage patients, the acute post-treatment elevation in heart rate was more pronounced in those who experienced greater clinical improvement, suggesting it could serve as a biomarker for identifying potential responders (Tan et al., 2024). Furthermore, in primary insomnia, HRV parameters and fractional amplitude of low-frequency fluctuations (fALFF) values in the sensorimotor network during continuous taVNS state are inter-related with treatment efficacy and are potential biomarkers for predicting patient response (Wu et al., 2021). The exploration of inflammatory biomarkers is also promising. In acute ischemic stroke, taVNS safely reduced post-stroke inflammation, and reductions in interleukin-6 (IL-6) levels correlated with improvements in neurological scores, indicating its potential as both a therapeutic mechanism and a response biomarker (Laurido-Soto et al., 2025a; Laurido-Soto et al., 2025b). In treatment-resistant schizophrenia, changes in the inflammatory cytokine TNF-α were correlated with clinical improvement following taVNS (Baig et al., 2026). These findings collectively underscore the potential of multi-modal biomarker approaches—encompassing physiological (pupil, HRV), neuroimaging (fMRI, EEG), and molecular (cytokines) measures—to stratify patients and optimize taVNS therapy.

Long-term follow-up studies are essential for evaluating the sustained effects and disease-modifying potential of taVNS. While many trials assess acute or short-term outcomes, evidence on durability is accumulating. In a study on post-stroke dysphagia, the improvement in swallowing function observed immediately after a 3-week taVNS intervention was maintained at a 4-week follow-up assessment (Wang et al., 2023). For treatment-resistant schizophrenia with negative symptoms, the significant improvement observed after a two-week taVNS intervention was sustained at a two-week follow-up (Baig et al., 2026). In a pilot study on female Long COVID patients, cognitive and psychological benefits observed after a 10-day t-VNS intervention remained or even progressed at a 1-month follow-up, with improvements in fatigue reaching significance at this later time point (Zheng et al., 2024). These studies suggest that therapeutic effects can persist beyond the active stimulation period. Investigating long-term effects also involves understanding the impact of repeated stimulation protocols. A study comparing single versus repeated (three sessions) taVNS protocols found that repeated sessions increased HRV, cognitive performance, and sleep efficiency, indicating potential cumulative benefits (Le Roy et al., 2023). In subarachnoid hemorrhage patients, repetitive taVNS over days increased overall heart rate variability and parasympathetic activity compared to sham treatment, with the most pronounced increase observed 2–4 days after initial treatment (Tan et al., 2024). Furthermore, long-term follow-up is critical for assessing the disease-modifying potential, such as preventing relapse or progression. For instance, in migraine, a 4-week taVNS treatment not only reduced headache frequency and intensity but also modulated abnormal thalamocortical connectivity, suggesting a possible impact on the underlying neural circuitry (Zhang et al., 2021). The protocol for an 8-week trial combining taVNS with aerobic training in hypertensive individuals includes a 30-day post-treatment assessment to evaluate sustained effects on blood pressure and autonomic function (Souza et al., 2026). Similarly, a proposed randomized controlled trial for postoperative aortic dissection patients plans follow-up assessments until 24 weeks post-surgery to evaluate the long-term impact of taVNS on gut-brain axis regulation and complications (Ning et al., 2025). These approaches highlight the growing recognition that determining the optimal treatment duration, understanding the persistence of effects, and evaluating potential long-term neuroplastic or immunomodulatory changes are fundamental to establishing taVNS as a viable long-term therapeutic strategy for chronic conditions.

### 7.3 Individual differences and personalization of treatment protocols

*Age, gender, baseline autonomic nervous system status, disease subtype, and even auricular anatomical variations can lead to significant individual differences in response to taVNS*.

Significant inter-individual variability in the efficacy of taVNS is a well-documented phenomenon, influenced by a complex interplay of demographic, physiological, and pathological factors. Age and gender are fundamental biological variables that can modulate treatment outcomes. For instance, a study in patients with minimally conscious state (MCS) suggested that earlier intervention might yield greater benefits, indirectly hinting at the potential influence of disease chronicity and possibly age-related neural plasticity (Zhou et al., 2023). Gender-specific effects are also plausible, as evidenced by a study exclusively enrolling female patients with fibromyalgia to investigate taVNS effects, acknowledging the higher prevalence in women and the potential for sex-specific pathophysiological and treatment response mechanisms (Akkurt et al., 2025). The baseline state of the autonomic nervous system (ANS) is a critical determinant of taVNS response. Research consistently shows that individuals with lower baseline parasympathetic tone, often indicated by reduced heart rate variability (HRV), may exhibit a more pronounced shift toward parasympathetic activation following taVNS. For example, in tinnitus-related mental stress, test-taVNS was most effective at improving parasympathetic function in patients with a low starting HRV level (Ylikoski et al., 2020). Similarly, in healthy individuals, a higher baseline low-frequency to high-frequency (LF/HF) ratio was associated with a greater decrease in this sympathetic-vagal balance metric after taVNS (Geng et al., 2022a). Disease heterogeneity further complicates the response landscape. In migraine without aura, the pretreatment functional state of brain networks involved in pain processing and modulation predicted clinical outcomes (Feng et al., 2022). For primary insomnia, the baseline functional activity of the default mode and visual networks was correlated with treatment efficacy, suggesting that specific neural substrates define responsive patient subtypes (Wu et al., 2025). Even within a single condition like primary dysmenorrhea, baseline electroencephalography (EEG) microstates related to salience and attention switching were found to mediate the relationship between baseline symptom severity and interindividual variability in taVNS efficacy (Wang C. et al., 2026). Furthermore, anatomical variations in the auricular innervation by the vagus nerve mean that identical electrode placements may not stimulate equivalent neural populations across individuals. This is supported by preclinical work showing that while cervical VNS and taVNS can produce similar overall activation in brainstem nuclei like the nucleus tractus solitarius, individual neurons show different activation profiles, indicating distinct afferent pathways (Owens et al., 2024). This inherent biological diversity underscores the challenge of a one-size-fits-all approach and necessitates a personalized framework for taVNS application.

*Research is dedicated to establishing algorithm models based on multimodal data (genetic, imaging, physiological) to recommend optimal stimulation targets, parameters, and treatment duration for individual patients*.

To address the challenge of inter-individual variability, contemporary research is increasingly focused on developing predictive models using multimodal biomarkers to guide personalized taVNS protocols. Neuroimaging, particularly functional magnetic resonance imaging (fMRI), has emerged as a powerful tool for identifying neural signatures predictive of treatment response. In migraine without aura, a support vector regression model using pretreatment fractional amplitude of low-frequency fluctuation (fALFF) features from regions within the thalamocortical circuits, default mode network, and descending pain modulation system successfully predicted the efficacy of a 4-week taVNS treatment (Feng et al., 2022). Similarly, for primary insomnia, the mean amplitude of low-frequency fluctuations (mALFF) in the default mode network (DMN) and visual network (VN) before treatment correlated with taVNS efficacy, and the combination of DMN and VN activity showed promise as a predictive biomarker (Wu et al., 2025). Physiological measures, especially those related to ANS function, are also key components of these models. In primary insomnia, heart rate variability (HRV) parameters during continuous taVNS, such as the root mean square of successive differences (RMSSD) and high-frequency (HF) power, were higher in treatment responders and correlated with both efficacy and baseline brain activity, suggesting an integrated brain-autonomic biomarker profile (Wu et al., 2021). EEG-derived metrics offer another rich data source. Microstate analysis of resting-state EEG revealed that baseline configurations could explain variability in taVNS efficacy for primary dysmenorrhea (Wang C. et al., 2026). Furthermore, EEG coherence in specific frequency bands, such as beta-band coherence between left frontal and parietal regions, has been correlated with clinical improvement in negative symptoms of treatment-resistant schizophrenia following taVNS, highlighting its potential as a sensitivity biomarker (Cui et al., 2025). The integration of such diverse data streams—genetic, structural and functional neuroimaging, electrophysiological, and autonomic—is the next frontier. The concept is to feed these multimodal datasets into machine learning algorithms to generate patient-specific recommendations. This could encompass the optimal stimulation site (e.g., cymba conchae vs. tragus, left vs. right ear), which has been shown to affect outcomes in conditions like metabolic syndrome and autonomic responses in healthy rats (Barbetti et al., 2025; de Moraes et al., 2023). It also includes parameter optimization (frequency, intensity, pulse width), as responses can be frequency-dependent, as seen in migraine where 1 Hz and 20 Hz taVNS modulated functional connectivity differently (Sacca et al., 2023), and intensity-dependent, as shown in studies on pupil dilation (Capone et al., 2021) and neuronal firing in rats (Owens et al., 2024). Finally, these models could inform treatment duration and scheduling, moving beyond fixed protocols to adaptive strategies based on continuous biomarker feedback.

*Exploring the optimal treatment model combining “on-demand stimulation” and “maintenance stimulation.”*

The exploration of optimal taVNS delivery paradigms is evolving beyond continuous, fixed-duration sessions toward more dynamic models that distinguish between acute, “on-demand” intervention and chronic, “maintenance” therapy. This distinction is crucial for tailoring treatment to the temporal dynamics of symptoms and underlying pathophysiology. “On-demand” or phasic stimulation refers to short bursts of taVNS delivered in response to specific triggers or during symptom exacerbation. This approach is particularly relevant for paroxysmal conditions or for modulating transient cognitive states. For instance, phasic, event-related taVNS has been shown to improve cognitive control, modulate pupillary responses, and alter low-frequency oscillatory power during an emotional Stroop task, demonstrating its potential for acute neuromodulation (Wienke et al., 2023). Similarly, short bursts of taVNS were found to enhance evoked pupil dilation in a dose-dependent manner, supporting its use for eliciting phasic noradrenergic responses (D'Agostini et al., 2023). In the context of acute stress, taVNS was shown to attenuate the initial heart rate increase associated with a cold pressor test, suggesting a role in buffering acute sympathetic arousal (Austelle et al., 2024). This model aligns with the concept of “closed-loop” taVNS, where stimulation is gated by physiological signals. Early forms include respiratory-gated auricular vagal afferent nerve stimulation (RAVANS), and future possibilities involve EEG- or ECG-gated systems that deliver stimulation synchronized with specific brain or cardiac states to maximize efficacy and efficiency (Yu et al., 2021). In contrast, “maintenance stimulation” involves regular, often daily, sessions over extended periods to produce sustained therapeutic effects, likely through induction of neuroplasticity, modulation of chronic inflammatory tone, or rebalancing of autonomic function. Chronic, intermittent low-level tragus stimulation over 6 months significantly reduced atrial fibrillation burden and inflammatory markers compared to sham, exemplifying the success of a maintenance paradigm for a chronic condition (Stavrakis et al., 2020). Likewise, a 4-week regimen of taVNS improved consciousness in patients with minimally conscious state, with benefits sustained at follow-up (Zhou et al., 2023). For irritable bowel syndrome-constipation, a 4-week taVNS protocol improved symptoms, rectal sensation, and autonomic markers (Liu et al., 2024b). The optimal therapeutic model likely involves a combination of both strategies: maintenance stimulation to establish a foundational improvement in disease state or neural network function, supplemented with on-demand stimulation to manage breakthrough symptoms or acute exacerbations. For example, in managing chronic pain conditions like fibromyalgia, a maintenance protocol could be used to reduce overall pain sensitivity and inflammation, while on-demand stimulation could be employed during acute pain flares. Future research must systematically compare the efficacy, parameter sets, and biomarker profiles of these different dosing regimens to establish guidelines for their combined use, ultimately moving toward fully personalized and adaptive taVNS therapy protocols. However, it is also important to acknowledge the heterogeneity and negative findings in the taVNS literature. Many studies lack sufficient statistical power due to small sample sizes, increasing the risk of false positives or inflated effect sizes. Publication bias may favor positive results, leaving null findings unpublished. Stimulation parameters vary widely across studies without systematic optimization, making negative results difficult to interpret. Patient characteristics—including disease subtype, baseline autonomic tone, and medication status—substantially influence treatment responsiveness. Future trials should adopt standardized protocols, report both positive and negative outcomes, and conduct subgroup analyses to identify which patients are most likely to benefit from taVNS.

### 7.4 Practical recommendations for future trial design

Based on the challenges discussed above, future taVNS trials should: (1) fully report all stimulation parameters; (2) use rigorous, sensory-matched sham controls and assess blinding success; (3) stratify by baseline autonomic status, age, and disease subtype; (4) incorporate validated biomarkers alongside clinical endpoints; (5) include long-term follow-up for chronic indications; and (6) preregister protocols following CONSORT guidelines.

## 8 Conclusion

In conclusion, taVNS is a promising non-invasive neuromodulation technique with preliminary efficacy in epilepsy, depression, chronic pain, inflammation, and cardiovascular diseases. However, several translational gaps remain, including parameter standardization, biomarker validation, and robust sham-controlled trials. First, parameter standardization would benefit from more consistent reporting across studies, as current protocols vary considerably in frequency, intensity, and duration, which limits cross-study comparison. Second, biomarker validation may help identify responsive patient subgroups and support a more personalized approach. Third, well-designed sham-controlled trials with adequate sample sizes and long-term follow-up are needed to further confirm efficacy and inform evidence-based guidelines. Mechanistically, taVNS acts through an integrated “ear-brain” pathway and the cholinergic anti-inflammatory axis, engaging autonomic, neuroplastic, and gut-brain mechanisms. This review provides two distinctive contributions: a convergent mechanistic framework and a critical identification of translational bottlenecks. Addressing these gaps would help advance taVNS from a research tool toward broader clinical application.
